# Divide and Conquer: Sub-Grouping of ASD Improves ASD Detection Based on Brain Morphometry

**DOI:** 10.1371/journal.pone.0153331

**Published:** 2016-04-11

**Authors:** Gajendra J. Katuwal, Stefi A. Baum, Nathan D. Cahill, Andrew M. Michael

**Affiliations:** 1 Autism and Developmental Medicine Institute, Geisinger Health System, Danville, PA, United States of America; 2 Chester F. Carlson Center for Imaging Science, Rochester Institute of Technology, Rochester, NY, United States of America; 3 School of Mathematical Sciences, Rochester Institute of Technology, Rochester, NY, United States of America; 4 Faculty of Science, University of Manitoba, Winnipeg, Canada; The George Washington University, UNITED STATES

## Abstract

Low success (<60%) in autism spectrum disorder (ASD) classification using brain morphometry from the large multi-site ABIDE dataset and inconsistent findings on brain morphometric abnormalities in ASD can be attributed to the ASD heterogeneity. In this study, we show that ASD brain morphometry is highly heterogeneous, and demonstrate that the heterogeneity can be mitigated and classification improved if autism severity (AS), verbal IQ (VIQ) and age are used with morphometric features. Morphometric features from structural MRIs (sMRIs) of 734 males (ASD: 361, controls: 373) of ABIDE were derived using FreeSurfer. Applying the Random Forest classifier, an AUC of 0.61 was achieved. Adding VIQ and age to morphometric features, AUC improved to 0.68. Sub-grouping the subjects by AS, VIQ and age improved the classification with the highest AUC of 0.8 in the moderate-AS sub-group (AS = 7–8). Matching subjects on age and/or VIQ in each sub-group further improved the classification with the highest AUC of 0.92 in the low AS sub-group (AS = 4–5). AUC decreased with AS and VIQ, and was the lowest in the mid-age sub-group (13–18 years). The important features were mainly from the frontal, temporal, ventricular, right hippocampal and left amygdala regions. However, they highly varied with AS, VIQ and age. The curvature and folding index features from frontal, temporal, lingual and insular regions were dominant in younger subjects suggesting their importance for early detection. When the experiments were repeated using the Gradient Boosting classifier similar results were obtained. Our findings suggest that identifying brain biomarkers in sub-groups of ASD can yield more robust and insightful results than searching across the whole spectrum. Further, it may allow identification of sub-group specific brain biomarkers that are optimized for early detection and monitoring, increasing the utility of sMRI as an important tool for early detection of ASD.

## Introduction

Autism Spectrum Disorder (ASD) is a group of developmental disabilities that manifest in early childhood. ASD is characterized by impaired communication and social skills, repetitive behaviors and fixated interests [1]. It is highly heterogeneous in its etiology, comorbidity, pathogenesis, genetics and severity [2–5]. Currently ASD diagnosis is primarily based on assessing the behavioral and intellectual abilities of a child. This diagnosis procedure can be subjective, time consuming, and inconclusive due to factors such as comorbidity [6]. Further, since it is based only on behavioral symptoms, it does not provide insight on the underlying etiology and cannot be utilized for early diagnosis and intervention.

Structural magnetic resonance imaging (sMRI) is a non-invasive tool and is used to capture the brain morphometry. ASD diagnosis based on sMRI can be objective and can be utilized even at the prenatal and neonatal stage [7]. Brain biomarkers derived from sMRI, in conjunction with other biomarkers, have been successfully utilized for early diagnosis of brain disorders such as Alzheimer’s [8–10]. The successful identification of brain biomarkers of ASD can help characterize the neuroanatomical basis of the ASD heterogeneity in etiology and pathogenesis. With successful identification of brain biomarkers, sMRI can also be a powerful technique for early diagnosis and intervention.

Characterizing ASD in terms of brain morphometry is challenging due to its heterogeneity [11]. However, a number of studies have investigated brain anatomical abnormalities in ASD compared to that of Typically Developing Control (TDC) subjects. The anatomical differences reported in previous studies are mostly contained in the frontal lobe, parietal lobe, temporal lobe, limbic system and cerebellum [12], but previously reported regions have not been consistent across studies [13–15]. The variability in differences can be due to the heterogeneity of ASD, differences in methodological approaches, or a combination of both.

Mass univariate techniques such as Voxel Based Morphometry (VBM) are widely applied in brain imaging studies to detect group level brain anatomical differences. Univariate approaches generally have high exploratory power but are fundamentally limited to the detection of inter-variable relationships. Multivariate pattern recognition techniques (MVPT) [16] can perform better on capturing the brain morphometry of a heterogeneous condition like ASD. MVPTs are capable of detecting subtle and spatially distributed differences in data and thus hold promise for characterizing ASD and its sub groups based on brain morphometry. In recent years, there have been a number of studies applying MVPT to sMRI features, particularly in ASD vs. TDC classification setting. These studies can be broadly categorized into two groups based on the number of subjects used in the study: (1) small dataset (n < 200) matched for demographics and behavioral (DB) measures such as age, sex and IQs [17–21] and (2) large heterogeneous datasets such as the Autism Brain Imaging Data Exchange (ABIDE) dataset (n > 700) [22–24]. Table 1, below, shows classification accuracies from previous studies and the disparity of results is clear. For instance, Group 1 using small datasets reports high classification accuracies while Group 2 using the large heterogeneous ABIDE dataset reports classification accuracies less than 60%.

**Table 1:** Previous ASD vs. TDC Classification studies.

Study	#ASD/#TDC	sMRI Features	Classification Technique	Classification Accuracy (%)
Group 1 (small dataset; n<200)				
Ecker et al. 2010	22/22 (Males)	Gray matter	SVM	81
Ecker, Marquand, et al. 2010	20/20 (right-handed)	Cortical thickness in left hemisphere	SVM	90
Jiao et al. 2010	22/16	Regional cortical thickness	LMT	87
Uddin et al. 2011	24/24	Gray matter in default mode network regions	SVM	90
Wee et al. 2014	58/59	Regional and inter-regional cortical and subcortical features	SVM	96
Group 2 (large dataset; n>700)				
Haar et al. 2014	539/573	Regional volume, surface area and cortical thickness	LDA, QDA	<60
Sabuncu & Konukoglu 2014	325/325	Regional volume, surface area and cortical thickness	SVM, NAF, RVM	<60
Katuwal et al. 2015	373/361	Volume, surface area, cortical thickness, thickness std., mean curvature, Gaussian curvature, folding index	RF, GBM, SVM	60

*SVM*: Support Vector Machine; *LMT*: Logistic Model Trees; *LDA*: Linear Discriminant Classifier; Q*DA*: Quadratic Discriminant Classifier; *NAF*: Neighborhood Approximation Forest; *RVM*: Bayesian Relevance Vector Machine; *RF*: Random Forest; *GBM*: Gradient Boosting Machine

In this study, we investigate the heterogeneity in ASD brain morphometry as a major reason behind the inconsistent neuroanatomical findings and the disparity in classification accuracies. We investigate if demographics and behavioral measures (DB) of the subjects can be utilized to mitigate the ASD heterogeneity and, hence help understanding ASD brain abnormalities and predicting ASD using brain morphometry. We use multiple automatically extracted brain morphometric features and multiple classification techniques for this investigation. First, we investigate the incremental predictive power that can be gained by adding DB measures such as autism severity (AS), verbal IQ (VIQ) and age to the brain morphometric features derived from sMRI. Second, we investigate if sub-grouping the subjects by the above mentioned DB measures helps to improve ASD vs. TDC classification. Third, we explore the important features for classification in the sub-groups and how they change with DB measures. Further, we try to explain the discrepancies in the reported neuroimaging findings on brain abnormalities in ASD and classification accuracies in relation to the heterogeneity in ASD brain morphometry. Finally, we discuss ASD heterogeneity in brain morphometry and future directions for neuroimaging studies to tackle it.

## Methods

### SMRI Data

The ABIDE [25] dataset with 1,112 sMRIs from 17 different sites was used in this study. Local Institutional Review Boards (IRB) approved the acquisition of the data in all sites. Consent to participate was obtained via IRB approval or explicit waiver. All data were fully anonymized as required by HIPAA guidelines. Each sMRI was visually inspected and the sMRIs with significant motion or other artifacts were excluded from analysis. Seventy-six subjects from two ABIDE sites were excluded due to poor image quality (motion/artifacts). An additional 96 subjects were excluded due to poor image quality, and another 64 subjects were discarded due to FreeSurfer [26] segmentation failure. Since ASD is highly prevalent in males [27] and also to avoid the gender effects, 142 female subjects were excluded. Finally, 734 male subjects (ASD: 361, TDC: 373) were used from the remaining 876 subjects. Summary of DB measures of the used sample is presented in Table 2. Scanner information of the individual sites can be obtained from Table 1 in [22].

**Table 2:** Subject demographics and behavioral (DB) measures.

	ASD	TDC	ASD vs. TDC t-test P-value
**N**	361	373	
**Age (years)**	17.9 ± 8.7 (7 to 64)	18.1 ± 8.2 (6.47 to 56.2)	0.7
**VIQ**	104.5 ± 17.8	112.4 ± 12.9	6.2E-10*
**PIQ**	105.2 ± 16.8	108.7 ± 13.2	5.7E-3*
**FIQ**	105.2 ± 16.6	111.8 ± 12.3	4.8E-9*
**ADOS**	11.9 ± 3.7	NA	NA
**AS**	7.1 ± 2.1	NA	NA

*VIQ*: Verbal IQ; *PIQ*: Performance IQ; *FIQ*: Full IQ, *ADOS*: Autism Diagnostic Observation Schedule; *AS*: Autism Severity

### Reasons for using male subjects only

We decided to use only male subjects in this study for several reasons. First, we wanted to remove the gender effects on the ASD brain heterogeneity and focus only on the brain morphometry of ASD males. This decision was motivated by the previous findings that there are significant differences between the brain anatomy of ASD males and females. Brain abnormalities in female ASD subjects reported by studies using only female subjects [28,29] have a very small overlap with the abnormalities reported by the studies performing meta analyses of predominantly male subjects [30,31]. A recent study by Lai et al. [32] focusing on the brain anatomical differences of ASD males and females has reported that the neuroanatomy of adult ASD males and females differed and there was minimal spatial overlap in both grey and white matter. Second, compared to females, ASD is more prevalent in males [27]. In addition, among the 876 high quality sMRIs available from ABIDE, 84% (734) of them were of males.

### SMRI Preprocessing

The *recon-all* preprocessing workflow of FreeSurfer v. 5.3.0 [33–36] was used to extract brain morphometric features. Recon-all is a fully automated workflow that performs all the FreeSurfer cortical reconstruction and sub-cortical segmentation steps in a unified pipeline. It includes several processing stages such as motion correction, non-uniform intensity normalization, Talairach transform computation, intensity normalization, skull stripping, and cortical parcellation steps. Volume of 40 sub-cortical structures from *Aseg* atlas [35] and volume, surface area, Gaussian curvature, mean curvature, folding index, thickness mean and thickness standard deviation of 34 cortical structures from *Desikan-Killiany* atlas [37] were derived from each sMRI. In total, 538 brain morphometric features were derived for each subject. The volume features of each subject were normalized by Total Intracranial Volume (TIV) since percentage or relative brain volumes have been found to be more robust across scanner types and scanner drifts [38] and thus this should reduce sensitivity to the fact that the data were collected at multiple sites.

### Classification Algorithms

Random forest (RF) [39] classification models were trained using brain morphometric features for ASD vs. TDC classification. RF was used since it is inherently suitable for parallel processing, has very few hyper parameters to tune, does not require scaling the data, is theoretically resistant to overfitting, provides variable importance and has been found to be very good for a variety of datasets [39,40]. To avoid the results from being affected by model selection bias, we repeated the experiments using Gradient Boosting Machine (GBM) [41] classifier models and the results are presented as S1 and S2 Figs. For both RF and GBM, *scikit-learn 0*.*16*.*1* [42] was used. For all classifiers, hyper parameter tuning was performed with cross-validation and classification performance was estimated by 10-fold cross-validation. RF and GBM classification models are briefly explained below and are explained in detail in the cited publications.

#### Random Forest (RF)

RF is an ensemble of decision trees and its output class is the mode value of the output classes of the individual decision trees. The name ‘random’ comes from the fact that it uses a random subsample of predictors (or features) on each step of recursive data partitioning i.e. while growing a decision tree. The optimal number of predictors used to split a node of a decision tree was automatically estimated by performing grid search within *(√m -√m /2*, *√m + √m/2)* where *m* is the number of features. Gini impurity [43] was minimized while growing decision trees. Gini impurity is the probability that a randomly chosen sample would be incorrectly labeled if the samples were labeled according to the distribution of class labels. In general, the higher the number of decision trees in a RF, the more reliable is the prediction and the interpretability of the variable importance [44]. So, a large number (5000) of decision trees were used in the RF classification models used in this study.

#### Gradient Boosting Machine (GBM)

A general boosting technique iteratively combines several weak or base learners into one strong learner. Here a learner means a basic prediction model such as decision tree. At each iteration or boosting step, GBM constructs a new base learner to be the most parallel to the negative gradient of a loss function along the observed data so that the new base learner focuses on the weakness of the model [45]. In other words, it performs functional approximation of a model by consecutively improving along the negative direction of a loss function. In this study, a binomial loss function was used and decision trees were used as base learners. A grid search on the depth of decision tree (1 to 12) and the subsample ratio (0.5 and 0.7) was performed to automatically estimate their optimum values. To increase the stability of the model and increase the reliability of the variable importance, a large number (5000) of decision trees were used in the GBM classification models used in this study. To avoid overfitting, a very low learning rate (0.001) was used.

### Metrics Used

Classification accuracy and area under the ROC curve (AUC) were used to measure the success of classification. Accuracy is a threshold-based metric and AUC is a ranking based metric. AUC is the probability that a classifier will rank a randomly chosen positive example higher than a randomly chosen negative example with the assumption that the positive example ranks higher than the negative example [46]. The practical difference or effect size of ASD vs. TDC group difference was quantified using Cohen’s d [47]. Cohen’s d is the standardized difference between two means defined as *(mean*_*1*_*-mean*_*2*_*)/SD*_*pooled*_ where *SD*_*pooled*_ is the weighted average of the standard deviations of two groups.

### Adding AS, VIQ and age information to the brain morphometric features

We reduced the heterogeneity of subjects by adding DB measures (AS, VIQ and age) to brain morphometric features and we achieved this by following two schemes. First, VIQ and age were used as training features in conjunction with the morphometric features. In the second scheme, the subjects were sub-grouped by AS, VIQ and age into three sub-groups by each as defined in Table 3. TDC subjects were not used while sub-grouping by AS. AS = 5 was used as the threshold between low and mid AS sub-groups according to Table 2 in [48]. The AS = 8 was used as the threshold between mid and high AS sub-groups since the mean and median for AS ≥ 6 are 7.9 and 8 respectively. Only 167 ASD subjects who had AS information were used in the sub-groups by AS. In sub-grouping by VIQ, the threshold of VIQ = 90 instead of natural choice 85 (one standard deviation below the median 100) was used to divide the low and normal VIQ sub-groups because there were very few subjects with VIQ ≤ 85. Only 586 subjects (296 ASD, 290 TDC) who had VIQ information were used in the sub-groups by VIQ. In sub-grouping by age, the thresholds 13 and 18 years were chosen because they approximately reflect pre-puberty, adolescence and early adulthood, and also yielded in sub-groups with similar sample sizes. Subjects greater than 40 years were not used as they were very few in number (17).

**Table 3:** Sub-groups definition.

	Sub-groups	Definition	#ASD/#TDC	
			Up-sampling	Down-sampling
	*mild*	4 ≤AS ≤ 5	20/373	20/20
**AS**	*moderate*	6 ≤ AS ≤ 7	60/373	60/60
	*high*	8 ≤ AS ≤ 10	76/373	76/76
	*low*	75 ≤ VIQ ≤ 90	57/15	15/15
**VIQ**	*normal*	90 <VIQ < 115	146/142	142/142
	*high*	115 ≤ VIQ ≤ 150	93/133	93/93
	*young*	6 ≤ Age < 13	116/120	NA
**Age (years)**	*mid*	13 ≤ Age < 18	114/108	NA
	*old*	18 ≤Age ≤ 40	121/138	NA

*NA*: Not Applicable. Down-sampling was not performed in the sub-groups by age since the number of subjects were comparable and very few subjects had AS and VIQ to use for matching the subjects.

In each sub-group, ASD vs. TDC classification models were trained using morphometric features. The sub-grouping resulted in an unbalanced classification problem in sub-groups i.e. classes with uneven sizes [49]. This problem was addressed in two ways: up-sampling the smaller class in each training fold and down-sampling the larger class. AUC has been used to evaluate the performance of classification models in sub-groups since AUC is insensitive to the unbalanced classes [50].

## Results

### Classification using only brain morphometric properties

Applying RF on brain morphometric features, classification accuracy of 60% and AUC of 0.61 was achieved. Similar classification performance has been reported by previous studies using the ABIDE dataset: Katuwal et al. 2015 (60%), Haar et al. 2014 (<60%) and Sabuncu & Konukoglu 2014 (<60%).

### Age and VIQ used as training features in conjunction with brain morphometric features

We first sought to determine whether simply adding age or VIQ as training features would improve brain morphometric classification. When age was added to the brain morphometric features for training the classifier, AUC improved to 0.62. When VIQ was added, AUC improved to 0.66. When both age and VIQ were added, AUC improved to 0.68. In addition, site information was explicitly added to the morphometric features for training the classifier. One-hot coding method was used to represent the site information, i.e. for each scanning site, a binary feature was added with values of one for subjects from the scanning site and values of zero for other subjects. In total, 17 binary features for 17 sites representing the site information were added to the 538 morphometric features. After adding site information to the morphometric features for training, AUC did not improve and was same as that from using only the morphometric features.

### Sub-grouping subjects by AS, VIQ and age

#### Up-sampling smaller class in each training fold

**Fig 1:**
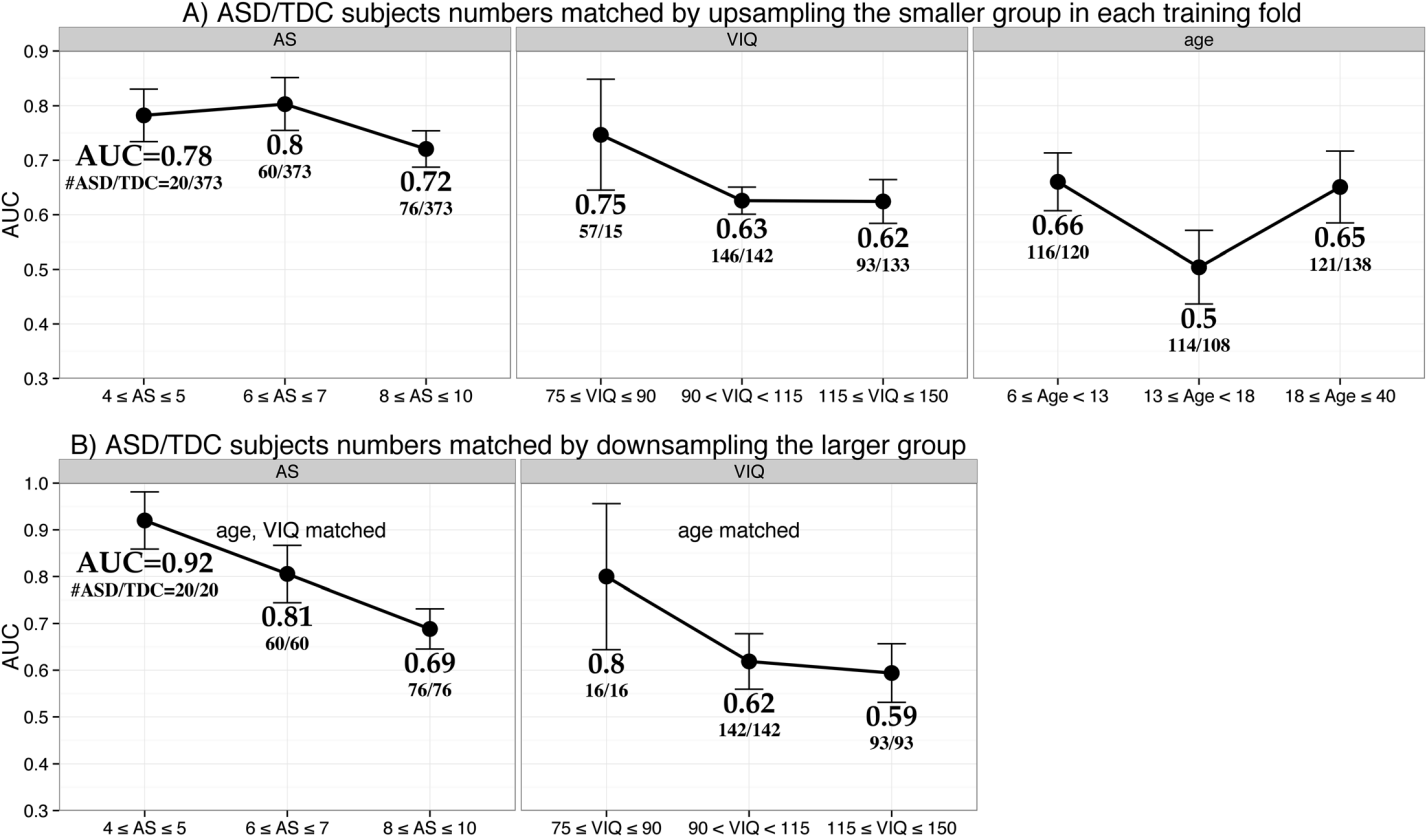
Improvement in classification by sub-grouping based on autism severity (AS), age and Verbal IQ (VIQ). The AUC scores of the classification in the sub-groups are presented. A point represents the mean and an error bar represents the one standard deviation of the AUC scores from 10 test folds. **A)** Smaller classes were up-sampled in each training fold to balance the number of ASD & TDC subjects. Sub-grouping improved the classification with the most and least improvements from sub-grouping by AS and age respectively. **B)** Larger classes were down-sampled matching the demographics of the smaller classes. This scheme further improved the classification performance.

In each sub-group, the smaller class was randomly up-sampled in each training fold to match the number of ASD and TDC subjects. The AUC scores achieved in the sub-groups are presented in Fig 1A and Table 4. In Fig 1A, a point represents the mean and an error bar represents the one standard deviation of the AUC scores from 10 test folds. AUC scores and number of ASD and TDC subjects are presented below the error bar.

**Table 4:** Classification AUC in sub-groups created by AS, VIQ and age.

		Up-sampling		Down-sampling	
		AUC		AUC	
	Sub-groups	RF	GBM	RF	GBM
	*mild*	0.78 ± 0.09	0.74 ± 0.16	0.92 ± 0.12	0.92 ± 0.11
**AS**	*moderate*	0.80± 0.10	0.76 ± 0.09	0.81 ± 0.12	0.80 ± 0.10
	*high*	0.72 ± 0.07	0.69 ± 0.07	0.69 ± 0.08	0.68 ± 0.11
	*low*	0.75 ± 0.20	0.71 ± 0.31	0.80 ± 0.30	0.80 ± 0.31
**VIQ**	*normal*	0.63 ± 0.05	0.65 ± 0.05	0.62 ± 0.11	0.63 ± 0.11
	*high*	0.62 ± 0.08	0.62 ± 0.07	0.59 ± 0.12	0.54 ± 0.13
	*young*	0.66 ± 0.11	0.67 ± 0.10	NA	NA
**Age (years)**	*mid*	0.50 ± 0.13	0.51 ± 0.14	NA	NA
	*old*	0.65 ± 0.13	0.66 ± 0.14	NA	NA

*NA*: Not Applicable. Down-sampling was not performed in the sub-groups by age since the number of subjects were comparable and very few subjects had AS and VIQ to use for matching the subjects. Mean and standard deviation of the AUC across 10 test folds are presented.

In sub-groups by AS, AUC was 0.78, 0.8 and 0.72 for low, moderate and high sub-groups respectively. The sample sizes in the sub-groups by AS were unequal. So, to check if the results were due to unequal sample sizes, separate classification models were built for the subjects with AS = 4–5 (#ASD/#TDC = 20/373), 6 (33/373), 7 (27/373), 8 (25/373), 9 (29/373) and 10 (22/373). The result of this experiment are presented in S5 Fig. In this experiment, where the sample sizes in the sub-groups were comparable, AUC decreased with the AS according to both RF and GBM. There was strong negative correlation (RF: r = -0.72, p = 0.1, GBM: r = -0.86, p = 0.028*) between mean AUC and mean AS of sub-groups; see S5 Fig.

In sub-groups by VIQ, AUC decreased with VIQ, with AUC of 0.75, 0.63 and 0.62 for low, normal and high VIQ sub-groups respectively. In sub-groups by age, AUC was modest in young and old sub-groups with AUC of 0.66 and 0.65 respectively. AUC was low (0.5) in mid-age sub-group.

In summary, sub-grouping the subjects by AS, VIQ and age improved the classification rate with the most and least improvements from sub-grouping by AS and age respectively. The results from GBM were similar and are presented in Table 4 and S1 Fig.

#### Down-sampling the bigger class to match the demographics of the smaller class

In the above section, although the subjects were more homogenous after sub-grouping, the distribution of other DB measures of ASD and TDC subjects in the sub-groups might be different. This raises a concern that the results from the up-sampling scheme could have been influenced by the difference in DB measures distribution. To check if the results are not due to the different demographics, ASD and TDC subjects in each sub-group were matched on demographics. In each sub-group, the bigger class was down-sampled to match the ASD and TDC subjects on age and/or VIQ; see Table 3 for the number of subjects. Subjects were matched by age and VIQ in the sub-groups by AS and by age in the sub-groups by VIQ. For sub-groups by age, down-sampling was not performed as the number of subjects in each group were comparable and very few subjects had AS and VIQ.

Classification performance further improved after matching the subject demographics. A high AUC of 0.92 was achieved in the low AS sub-group; see Table 4 and Fig 1B. Similarly, high AUCs of 0.81 and 0.80 were achieved for moderate AS and low VIQ sub-groups respectively. The AUC trends from this experiment were the same as that from the up-sampling scheme presented above, i.e. AUC decreases with AS and VIQ. The results from GBM were similar and are presented in Table 3 and S1 Fig. When separate classification models were built for the ASD subjects with each level of AS, AUC sharply decreased with AS according to both RF and GBM (RF: r = -0.86, p = 0.029*, GBM: r = -0.87, p = 0.026*); see S5 Fig.

To confirm that the increase in classification performance after sub-grouping is not due to optimization issues and is actually due to the reduction in the heterogeneity in the sub-groups, we tested the RF classification model trained in one sub-group on other sub-groups. To obtain the distribution of test score, the classification model trained in one sub-group was tested on 200 bootstrap replications from other sub-group. Classification results are presented in S3 Fig, where each sub-plot represents a sub-group and three data points correspond to the performance scores (when tested on the sub-group) of the models trained in three sub-groups. The AUC scores in intra-subgroup classification were much larger than the AUC scores in inter-subgroup classification in 16 out of 18 comparisons. AUC score of intra-subgroup classification was lower than inter-subgroup classification in 2 comparisons. This disparity occurred in the mid-age sub-group where the intra-subgroup classification was close to chance (50% success) and the inter-subgroup rates were also close to chance (53% and 52% success). Moreover, the AUC scores decreased when the difference between the training and testing sub-groups increased along the variable by which sub-groups were defined. For example, when the classification models were tested on the low-VIQ sub-group, the AUC scores decreased from 0.75, 0.64, and 0.35 respectively as the subjects from the low, mid, and high VIQ sub-groups were used for training the models.

### Multivariate analysis: Important features for classification and their variability across sub-groups

**Fig 2:**
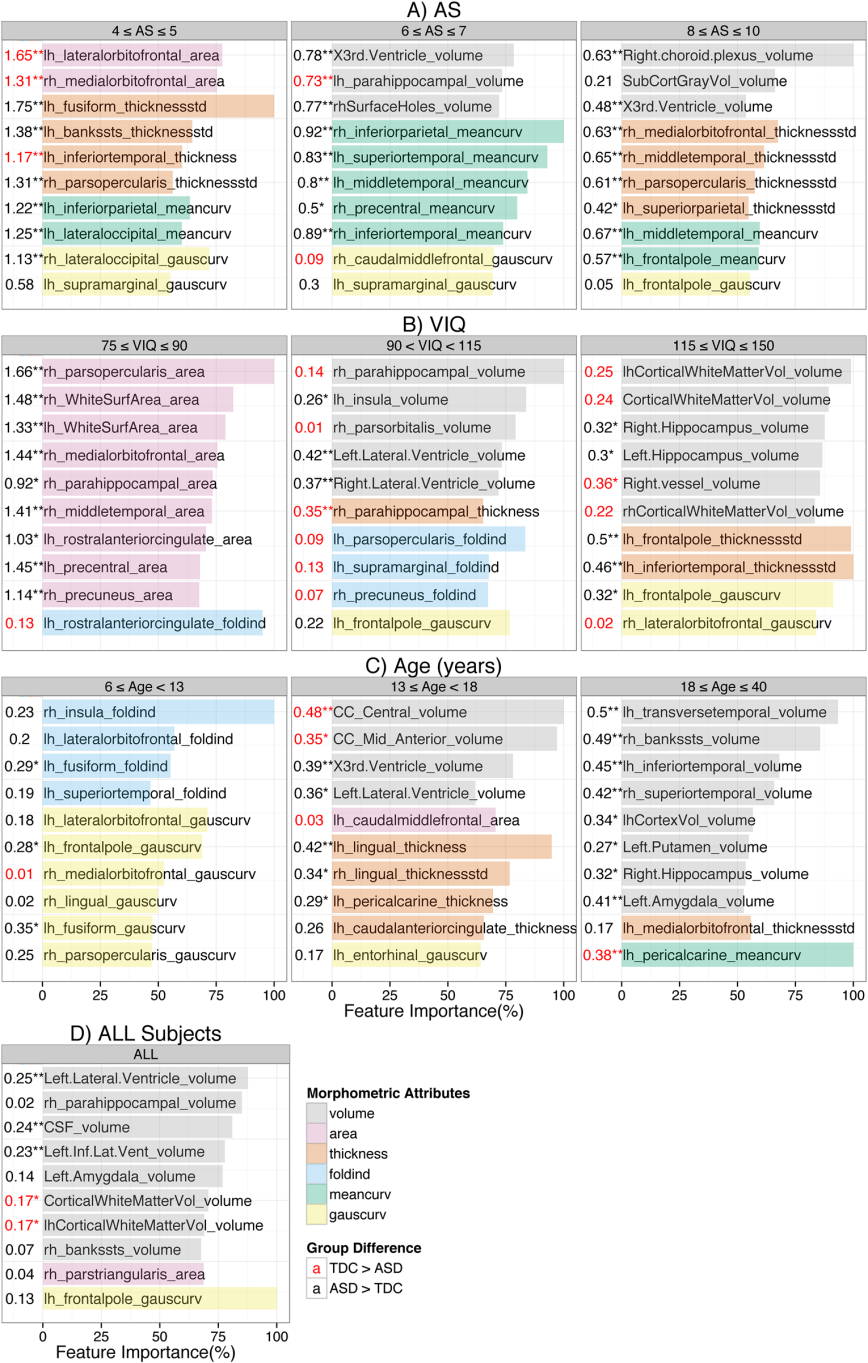
Important features for classification are different across sub-groups. Top 10 important features for autism spectrum disorder (ASD) vs. typically developing controls (TDC) classification in each sub-group (by AS, VIQ, age) are presented. Each feature is represented by a colored bar; the length of the bar represents the relative % importance for classification with respect to the top feature. The features have been grouped and color-coded by volume, area, thickness mean, thickness standard deviation, folding index, mean curvature and Gaussian curvature. Before each feature, Cohen’s d and two sample t-test significance (*P*<0.005** and *P*<0.05*) of ASD vs. TDC group difference are presented. The important features for classification varied across the sub-groups demonstrating the heterogeneity in ASD brain morphometry.

The top 10 important features for classification in each sub-group with matched subjects (i.e. from section 4.3.2) are presented in Fig 2. The top features for classification across all subjects are in Fig 2D. Each feature is represented by a bar whose length is proportional to its importance for the classification. The feature importance was calculated as an average of the importance scores from 10 test folds. Before each feature, ASD vs. TDC Cohen’s d and two sample t-test significance (P<0.005** and P<0.05*) are presented. The different morphometric features are color coded and have been grouped together. Findings for the volume features reported in this study are after they were normalized by TIV.

The important features for classification varied across the sub-groups. However, the important features were mainly from the frontal, temporal, insular, ventricular, right hippocampal and left amygdala regions. Most of the important features from RF and GBM were common; see Fig 2 and S2 Fig.

To remove the concern that the arbitrary cutoff of top 10 might have influenced our results, the important features were also selected by another technique based on cumulative distribution of the feature importance scores. After sorting the features in descending order of their importance scores, the scores were cumulatively added starting from the most important feature. The features required to reach the 10% of the total sum of the scores were considered important and the corresponding feature importance plot for RF is presented as S4 Fig. In addition, we relaxed our criteria for important features and used 25% threshold; see S5 Fig. Even after using this different technique to select the important features with multiple thresholds, the top features for classification were highly dissimilar across the sub-groups.

The important features according to two classifiers were similar suggesting that the results are not influenced by model choice. To statistically verify the similarity, we performed the Pearson’s correlation test between the importance scores of all features from the two classifiers. We performed the test separately in nine sub-groups and the correlation coefficients are reported in S1 Table. All correlation coefficients were high (r > 0.75 in 9 and r > 0.85 in 7 sub-groups) and statistically significant (p < E-16). The high similarity between the feature importance scores from two different classifiers supports that the important features reported in this study are not affected by model choice and hence are robust.

**Fig 3:**
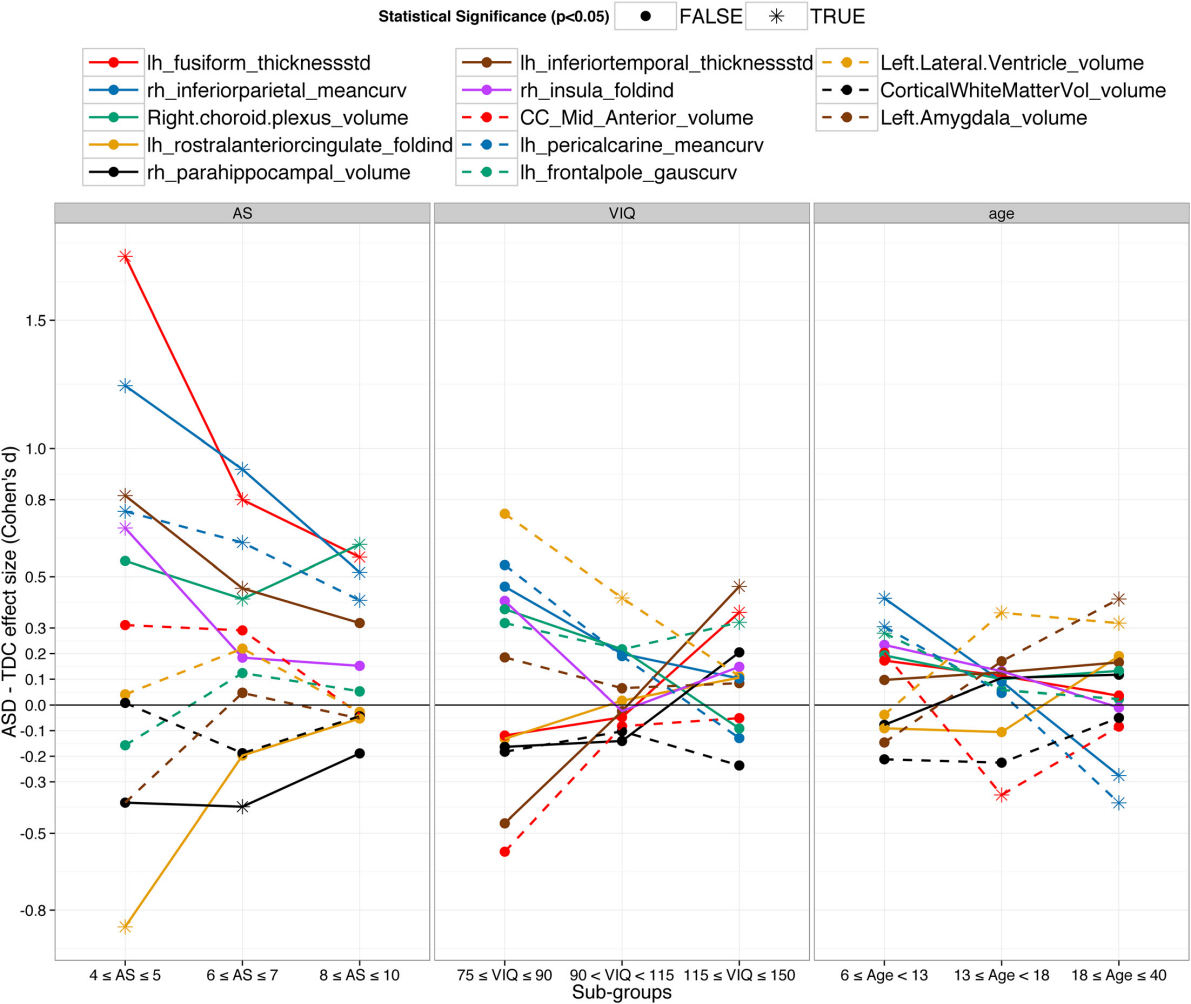
Variability of the important features with autism severity (AS), verbal IQ (VIQ) and age. A total of 13 important features for classification are presented; the top feature for each sub-group (nine in total) and the four important features for all subjects. The magnitude and direction of the ASD vs. TDC group differences of the top features varied with AS, VIQ and age demonstrating the heterogeneity in brain morphometry.

To demonstrate the extent of the heterogeneity in brain abnormalities, variability of the 13 important features with AS, VIQ and age are presented in Fig 3. The 13 features include the top feature from each sub-group (nine in total) and four important features from the classification using all the subjects.

Curvature and thickness based features were predominant in the sub-groups by AS; see Fig 2A. Interestingly, there were no important volume features in the low-AS sub-group. Volume features were present in moderate and high-AS sub-groups and many of them were from ventricles. Thickness standard deviation of the left fusiform gyrus (red line in Fig 3) was the most important feature in the low AS sub-group and had very large ASD vs. TDC group difference (d = 1.75, p = 4E-16*). The group difference decreased with AS but was still high (d = 0.57, p = 0.0006) in the high AS sub-group. Interestingly, the group difference even changed its direction with VIQ. The difference was negative (ASD < TDC) with small effect size (d = -0.11, p = 0.7) in the low-VIQ sub-group but was positive and statistically significant with medium effect size (d = 0.36, p = 0.01*) in the high-VIQ sub-group. Similarly, mean curvature of the inferior parietal gyrus (blue line in Fig 3) was the most important feature in the moderate-AS sub-group. It was significantly larger in ASD (d = 0.91, 2E-6*). The group difference decreased with AS but was still high (d = 0.51, p = 0.0006*) in the high AS sub-group. This feature also showed the reversal in the direction of the group difference- ASD > TDC with medium effect size (d = 0.41, p = 0.02*) in the young-age sub-group and ASD < TDC with medium effect size (d = -0.3, p = 0.03*) in the old-age sub-group. Right choroid plexus volume (green line in Fig 3) was the most important feature in the high-AS sub-group and had positive (ASD>TDC) group difference with large effect size (d = 0.55, p = 9E-4*). Across all subjects, it was larger in ASD with small effect size (d = 0.18, p = 0.02*). There was large positive group difference (d = 0.71, p = 0.05) in the low-VIQ sub-group, however, it decreased with VIQ and was negative in the high-VIQ sub-group (d = -0.15, p = 0.3).

Folding index of left rostral anterior cingulate gyrus (orange line in Fig 3) was the most important feature in the low-VIQ sub-group with small negative group difference (d = -0.13, p = 0.7). It is an interesting observation that it is the most important feature for classification even when the ASD vs. TDC group difference is very small and statistically insignificant. One thing to remember is that it is the most important in the multi-variate setting where the importance of a feature is dependent on its relationship with other features. For example, the group difference of the ratio of folding index of left and right rostral anterior cingulate gyrus was large (d = 0.7, p = 0.05). This demonstrates the superiority of MVPTs over univariate techniques by its ability to automatically find inter-variable relationships important for inter-group distinction. Similarly, volume of right parahippocampal gyrus (black line in Fig 3) was the most important feature in the mid-VIQ sub-group with small negative group difference (d = -0.15, p = 0.2). It was also an important feature in classification using all subjects; see Fig 2D. Thickness standard deviation of left inferior temporal gyrus (brown line in Fig 3) was the most important feature in the high-VIQ sub-group where it was larger in ASD with medium effect size (d = 0.46, p = 0.002*). However, the group difference was nearly zero in the normal-VIQ sub-group and even flipped its direction in the low-VIQ sub-group (d = -0.45, p = 0.2).

The important features across the sub-groups by age were distinct. Folding index and Gaussian curvature features from the frontal and temporal regions were predominant and there were very few volume, thickness and area based important features in the young-age sub-group. The volume features became more dominant with increase in age—most of the important features in the old-age sub-group were volume-based. Folding index of right insula gyrus (purple line in Fig 3) was the most important feature in the low-age sub-group with small positive ASD vs. TDC group difference (d = 0.23, p = 0.08). The group difference decreased with age and was nearly zero for the old-age sub-group. Volume of the mid anterior corpus callosum (dotted red line in Fig 3) was the most important feature in the mid-age sub-group where it was smaller in ASD (d = 0.35, p = 0.03*). However, in the young-age sub-group, it was larger in ASD (d = 0.15, p = 0.4). Mean curvature of the left pericalcarine gyrus (dotted blue line in Fig 3) was the most important feature in the old-age sub-group and was larger in ASD (d = 0.3, p = 0.02*) but was smaller in ASD (d = -0.38, p = 0.002*) in the old-age sub-group.

Across all subjects, Gaussian curvature of frontal pole was the most important feature. The volume features were predominant and were mainly from the left amygdala, right parahippocampal, ventricular and temporal regions. As other important curvature based features, the group difference in Gaussian curvature of frontal pole (dotted green line in Fig 3) was the largest in younger subjects (d = 0.28, p = 0.03*) and was the smallest for older subjects (d = 0.02, p = 0.9). Among the ventricular volumes, left lateral ventricle volume (dotted orange line in Fig 3) was the most important across all subjects (d = 0.24, p = 0.002*). It was larger in ASD and the group difference decreased with VIQ and increased with age. Across all subjects, all the ventricles were larger in ASD compared to TDC and the group differences were statistically significant (before multiple comparisons). In general, except 3rd and 4th ventricles, the group difference in ventricles decreased with VIQ; see S5 Fig. Left amygdala (dotted brown line in Fig 3) was also an important feature for classification across all subjects. It was larger in ASD in the old-age sub-group with medium effect size (d = 0.41, p = 0.001*) but was smaller in the young-age sub-group (d = -0.15, p = 0.3). Likewise, most of the important features showed high variability with AS, VIQ and age and even changed the ASD vs. TDC group difference direction.

## Discussion

Modest classification success is achieved using only brain morphometric properties. We demonstrated that the low success rate was due to the heterogeneity in ASD abnormalities by showing the variability of important features across the sub-groups created by demographics and behavioral measures AS, VIQ and age. To mitigate the challenges imposed by the ASD heterogeneity, we then utilized AS, VIQ and age information in conjunction with the brain morphometric features. We were able to significantly improve the classification success after utilizing extra information from AS, VIQ and age and demonstrated this using two different classification techniques. When the classification models trained in one sub-group were tested on other sub-groups, the inter-subgroup classification scores were much lower than the intra-subgroup classification scores. Moreover, the classification scores decreased when the difference between the training and testing sub-groups increased along the variable by which sub-groups were defined These results support our hypothesis that the sub-grouping of subjects results in the heterogeneity reduction and hence the improvement in classification performance.

The analysis of the important features for classification in conjunction with the univariate tests provided valuable insight on structural abnormalities of autistic brains. The abnormalities were mainly from ventricular, frontal, temporal, left amygdala and right hippocampal regions of the brain. The important features from two different classification techniques were similar demonstrating the robustness of our results. Below we discuss some interesting observations on heterogeneity of ASD brain morphometry in relation to the previous inconsistent neuroanatomical findings and discrepancies in the classification accuracies. In addition, challenges and future directions for neuroimaging studies on ASD prediction using brain morphometry are discussed.

### Classification becomes difficult with increase in AS

Classification AUC was the lowest in the high-AS sub-group for both up-sampling and down-sampling schemes; see Fig 1. The results from GBM were similar and are presented in S1 Fig. Moreover, AUC decreased with AS when sub-groups were created for each AS values according to both RF and GBM classification models; see S6 Fig. This result is opposite to that of Katuwal [23] where it was reported that the classification accuracy increases with Autism Diagnostic Observation Schedule (ADOS) score. AS is the standardized version of ADOS [48]. This discrepancy is likely due to the difference in experiment design- the classification model was trained across all subjects in the experiment of Katuwal [23] and separate classification models were trained in each sub-group in this study. In addition, only 167 ASD subjects had AS score available and were present in AS sub-groups of this study, while in Katuwal [23], 361 ASD subjects were used to train classification models in leave one out cross validation framework.

The decrease in AUC suggests that the most severely autistic subjects are the most difficult to classify, perhaps because the most severely autistic subjects are the most heterogeneous. In each sub-group, to check the heterogeneity in ASD data, we calculated the net variance as mean of the relative standard deviation (σ/mean) of all features. The net variance in ASD brain morphometry increased with AS– 0.42, 0.47 and 0.49 for low, mid and high AS sub-groups respectively. Even in the down-sampling scheme with demographics matched subjects, the net variance of the brain morphometry of ASD subjects increased with AS- 0.46, 0.55 and 0.57 for low, mid and high AS sub-groups respectively. This suggests that the brain morphometry of ASD subjects becomes more dissimilar to each other with increase in their severity, and hence increasing the classification difficulty.

In this study, we are not suggesting that the predictive models constructed for each AS sub-group can be directly used in clinical practice. ASD subjects were sub-grouped by AS only to investigate how ASD vs. TDC classification success and the features important for classification change with AS. By demonstrating that the important features are very dissimilar across the severity scores, we are suggesting that stratifying ASD subjects by severity scores might be helpful to better understand the brain abnormalities in ASD subjects. In future clinical practice improved knowledge of brain abnormalities in ASD subjects will provide evidences to support currently existing clinical diagnosis that are based on behavior.

### Folding index and curvature features maybe important for early detection of ASD

**Fig 4:**
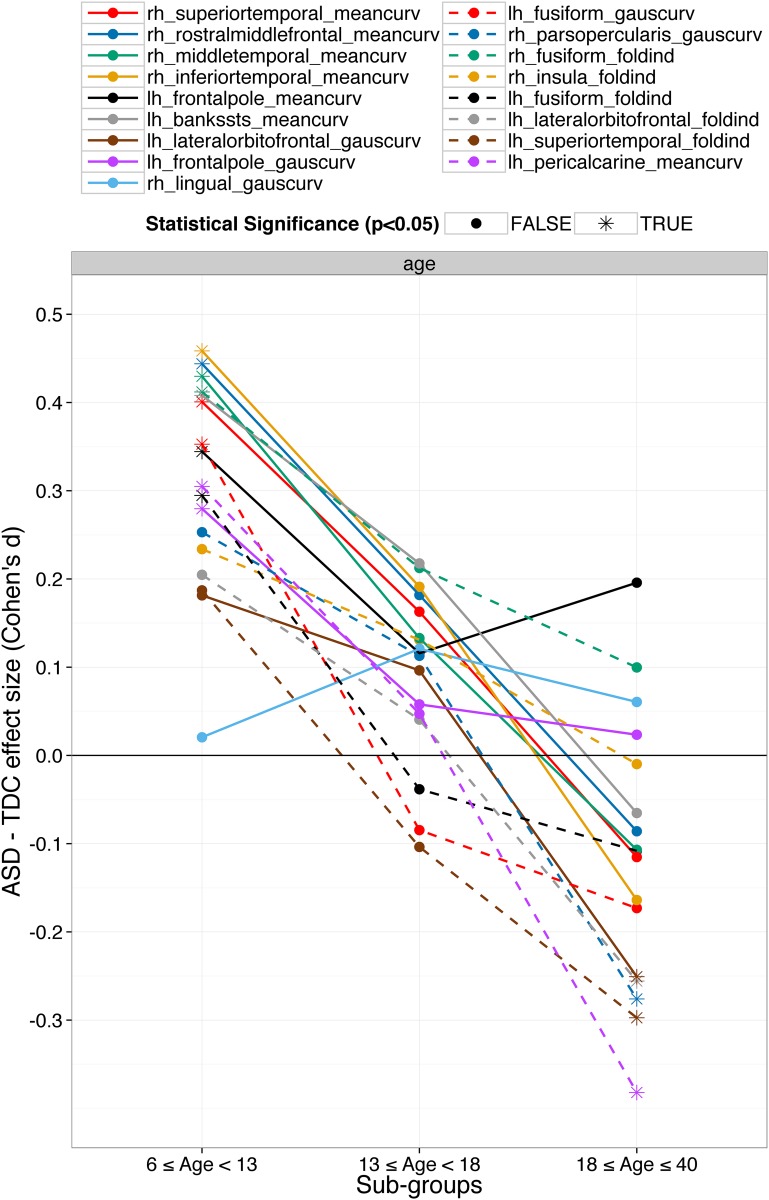
Folding index and curvature features are important for classification in young subjects i.e. in the young-age sub-group. The important folding index and curvature features from the young-age sub-group and/or whose ASD vs. TDC group differences across all subjects were statistically significant (multiple comparisons uncorrected) are presented. The features are mainly from frontal, temporal, lingual and insular region, and are larger in ASD. However, the group differences decrease with age and even the direction of the group difference flips for 12 out of 17 features.

In the young age sub-group, where the classification was performed between young ASD and young TDC subjects, most common top important features for classification were folding index and curvatures (mean and Gaussian) of frontal, temporal, lingual and insular regions. The importance of these features decreased with age and eventually in the old-age sub-group, volume features were predominant; see Fig 2C. The folding index and curvature features that were important in the young-age sub-group and/or whose ASD vs. TDC group differences across all 734 subjects were statistically significant (multiple comparisons uncorrected) are presented in Fig 4. All features (except Gaussian curvature of right lingual gyrus) were larger in young ASD subjects compared to young TDC subjects. The group difference decreased with age and the direction for 12 out of 17 features even flipped the direction (smaller in ASD) in the old-age sub-group. This result not only demonstrates the heterogeneity in ASD brain morphometric differences but also gives an important clue for the early diagnosis of ASD using brain morphometry. This result suggests that the most prominent brain morphometric abnormalities in young ASD subjects maybe the folding index and (mean and Gaussian) curvatures of the frontal, temporal, lingual and insular regions. Nordahl et al. [51] have also reported that cortical shape abnormalities (measured by sulcal depth) were more pronounced in the children (7.5–12.5 years). Similarly, Auzias et al. [52] have reported a statistically significant and consistent pattern of shape abnormalities in central, intra-parietal and frontal medial sulci in the children (18–108 months). They also reported that the shape descriptors of several sulci from frontal and temporal regions and age were statistically significant. Moreover, they also report significant correlations between the different sulcus shape descriptors and Childhood Autism Rating Scale (CARS) and ADOS scores.

Knowledge of brain morphometric differences in young ASD subjects is comparatively more valuable than that in old ASD subjects. Successful identification of robust brain biomarkers for ASD diagnosis in young patients would allow early intervention and hence likely increase the success of ASD treatment. Our results show that the shape abnormalities of frontal, temporal, lingual and insular brain regions are important to classify ASD from TDC in young children and hence the shape of these regions merit special attention. However, most of the previous studies on ASD brain abnormalities are based on volume, area and thickness features and very few are on shape features such as curvature, folding index and sulcal depth. We therefore emphasize the need to include less conventional features of brain morphometry like these, as well as others, in aiding classification of ASD.

### ASD heterogeneity in brain morphometry

We were able to increase the classification AUC up to 0.68 from 0.61 by adding AS, VIQ and age with the brain morphometric features. This shows that the brain morphometry and DB measures have some non-overlapping information. In addition, when the subjects were sub-grouped by AS, VIQ and age and the classification models were trained in each sub-group, there was significant improvement in classification performance. The important features for classification and the strength and the directionality of the ASD vs. TDC group difference in the important features highly varied across the sub-groups. This variability was presented in detail in section 4.4. Previous studies have also reported variability in brain abnormalities with factors such as age, gender, handedness etc. Lai et al. [32] reported that the brain regions affected in ASD males and ASD females have little overlap. Similarly, Floris et al. [53] reported strong rightward lateralization in the posterior and anterior mid-body of corpus callosum and found that the pattern of the lateralization is strongly depended on the handedness of the subjects with autism. A recent study by Lin [54] reported that the regional brain volume differences between ASD and TDC males are highly age-dependent. Through the age-stratified analyses, they showed that the patterns in GM and WM volumetric alterations in ASD are distinct among the subsamples of children, adolescents and adults. Therefore, the results from this study and the previous studies support the hypothesis that brain abnormalities in ASD are highly heterogeneous across the ASD population.

### Low classification success in large multi-site data due to ASD heterogeneity

The heterogeneous nature of the brain morphometry in ASD partially explains the discrepancy in the predictive performances reported by the two groups of previous studies: small sample size studies reporting high classification accuracies and the studies using the large multi-site ABIDE dataset reporting low classification accuracies. This is counterintuitive to the general idea that the generalized classification performance increases with the training sample size [55]. Large number of subjects from the multi-site datasets such as ABIDE provide more information about brain morphometry than that from small sample size. However, the amount of variance added due the ASD heterogeneity can surpasses the extra information gained with the increase in sample size. As the heterogeneity of the data increases, the training, validation and testing folds used for estimating generalized predictive performance of a model become more dissimilar to each other. As a result, the model trained using training and validation folds performs poorly in the test fold, hence decreasing the generalized predictive performance estimated by cross validation. This explains the low accuracies (< 60%) achieved by the previous studies using the ABIDE dataset. A much larger dataset is required so that the information gained from increase in sample size is greater than the increase in variance introduced by the heterogeneity of ASD. Larger standardized datasets easily accessible to the research community would, therefore, be highly valuable. On the other hand, subjects collected in a site and matched for factors such as age, sex, IQs etc. are relatively more homogenous. The training, validation and testing folds are more similar to each other, hence, the predictive performance of model estimated by cross validation is larger. This explains the high accuracies achieved by the previous studies using small well-matched data. In addition, although all previous studies using small sample size have reported the use of cross validation to estimate the predictive performance, many of them have not explicitly mentioned that the feature selection and classification steps were done under the same cross validation framework. When the two steps are under different cross validation framework i.e. when the feature selection is performed using all data, there will be data leak. The data leak results in an over-fitted model whose predictive performance cannot be generalized with confidence. A recent study by Katuwal [23] has reported that the high classification accuracies reported in some of the previous studies might be due to over-fitted models caused by data leak. In addition, the study demonstrated that the amount of over-fitting and hence the overestimated predictive performance increases with the decrease in sample size. Another reason for high classification accuracies in small datasets may arise due to manual feature engineering performed specific to the dataset. Caution should be taken to interpret the manually crafted features used in these studies. The interpretation of the important features for classification would be more relevant and meaningful with respect to the subjects’ DB measures such as age, sex, IQs, handedness etc.

### Future direction for neuroimaging studies

Developing a single successful classification model to predict autism type and severity using brain morphometry is likely the ultimate objective of the research on ASD diagnosis using brain morphometry. However, there are a few limitations and road blocks to be overcome before this objective can be achieved. First, it requires a much larger standardized data set than currently available datasets such as ABIDE. Second, based on the results shown here, it might not provide insight into the neuroanatomical basis of ASD as it would be difficult to perform exploratory analysis across large heterogeneous subjects compared to that in distinct homogenous sub-groups.

#### Divide and conquer: focus on smaller distinct homogenous sub-groups

**Fig 5:**
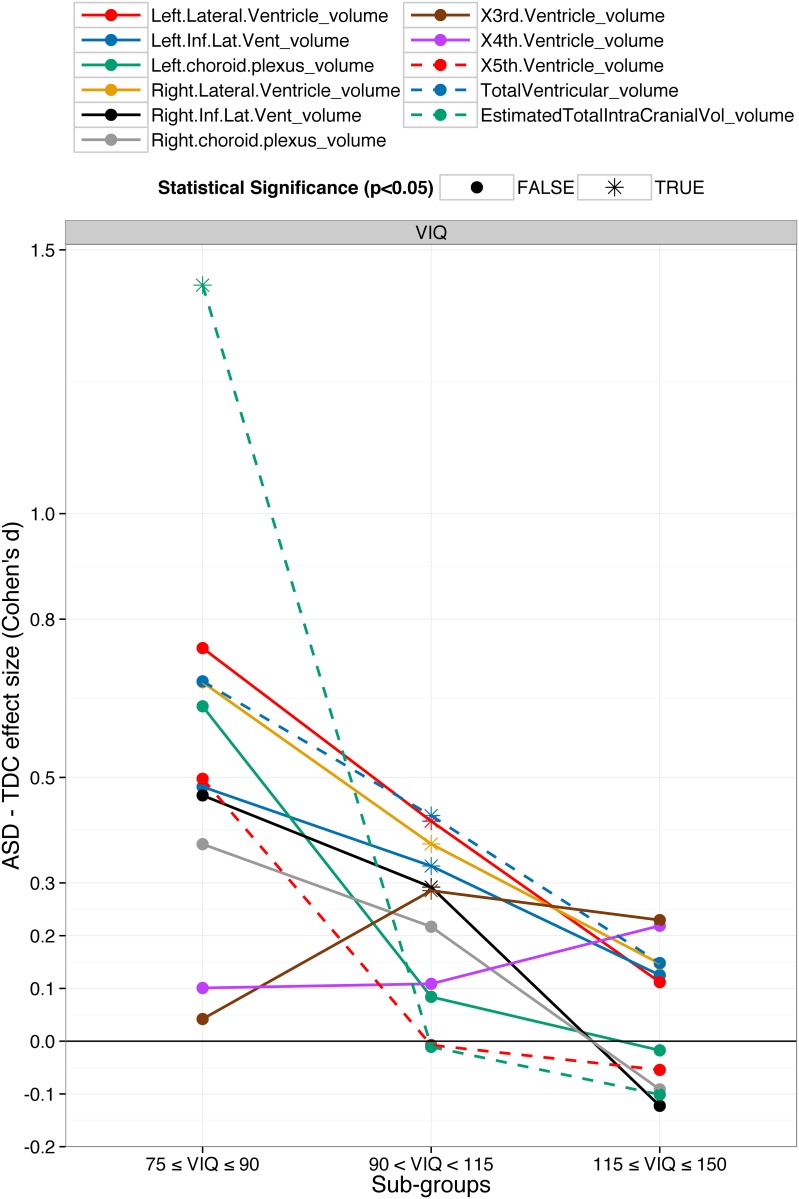
ASD vs. TDC group difference in ventricular volumes decreases with verbal IQ (VIQ). The ventricular volumes were normalized by total intracranial volume. Across all subjects, except 3^rd^ and 4^th^, all ventricles and total intracranial volumes were larger in ASD. When the subjects were sub-grouped by VIQ, the group differences were the largest in the low-VIQ sub-group but decreased with VIQ. For some ventricular, the direction of group difference even flipped in the high-VIQ sub-group i.e. volumes were larger in TDC for some ventricles.

ASD as currently diagnosed is a collection of autisms. It is highly heterogeneous in its etiology, comorbidity, pathogenesis, genetics, severity and brain morphometry [2–4,11]. As shown in Figs 3 and 4, brain abnormalities in ASD compared to TDC are highly variable across AS, VIQ and age. For highly heterogeneous conditions such as ASD, it is very hard to find a robust global brain biomarker. A better alternative may be to focus on the relatively more homogenous smaller sub-groups defined by a number of criteria such as age, sex, IQs, handedness, severity, etc. Several previous studies have suggested the same [11,56]. Dividing the ASD population in distinct sub-groups provides more exploratory power to the study and provides deeper insights into the anatomical abnormalities in ASD. The brain biomarkers identified by this technique albeit local with respect to some DB measure, are more robust and provide valuable insight on their effects in relation to the respective DB measure. For example, when the subjects were grouped by age and classification was done separately, folding index and curvature features were predominant in younger subjects (see Fig 3C and section 5.2). ASD detection in young age is more desirable as it would provide more time for early intervention. So for the classification in young subjects, one approach might be to focus on the folding index and curvature based features from insular, fusiform, frontal and temporal regions. Similarly, although the ventricular volumes were larger in ASD and were important features for classification, the ASD vs. TDC group difference sharply decreased with VIQ and, in subjects with high VIQ, the directionality of group difference even reversed for some ventricular volumes; see Fig 5. This result suggests that the ventricle volumes may be robust biomarkers in the low and normal VIQ population but certainly not for the high VIQ population. Thus brain biomarkers identified in distinct sub-groups will be more robust and insightful and hence more helpful to understand the neuroanatomical basis of the ASD heterogeneity.

In addition to the sub grouping of the ASD population, we propose adding the DB measures of the subjects with the sMRI features to train multivariate machine learning models. This data-driven automatic approach would help to identify the relationship of DB measures with the multi-variate brain morphometry and hence provide better insights on brain abnormalities in ASD. For example, identifying the robust relationship between age and multi-variate patterns in brain morphometry would be highly valuable to the understanding of the pathogenesis of ASD. Eventually, this could make sMRI a powerful tool for early detection of ASD.

#### Need for better features and better methods

The morphometric features estimated using the preprocessing tools may not accurately reflect the underlying morphology for several reasons. First, T1-weighted MRIs do not have enough information to distinguish between all the brain structures. For example, they do not have enough contrast between inter-sulcal CSF and skull. Second, preprocessing tools make a number of assumptions which do not always hold in all subjects, leading to erroneous estimates. For example, Morey et al. [57] showed that the correlation of the amygdala volume from manual tracing with the volumes estimated from FreeSurfer and FSL were only 0.56 and 0.24 respectively. This suggests that the uncertainty or the noise added during the automatic extraction of brain features is one of the major obstacles to automatic ASD detection using brain morphometry. So, the development of better automatic preprocessing tools is needed. In addition, preprocessing tools can be optimized to utilize data fusion techniques to decrease the uncertainty in the estimation of brain features. For example, T1 and T2-weighted images contain complementary information which can be utilized for more robust estimation of brain features.

In this study, we utilized regional-level features i.e. values describing the morphometric features of the brain regions. There may be fundamental limits to or an unpractical sample size required to achieve high classification performance using these features, suggesting future directions for work. Features from different spatial scales can be unified to improve the predictive performance of the classification using sMRI. In addition, shape features can be incorporated in the classification model. Moreover, hierarchical architecture such as convolution neural network (CNN) [58] can be applied for automatic identification and extraction of features. This may help to improve the predictive performance and may also provide better insights into the neuroanatomical underpinnings of ASD due to the hierarchical nature of its features. Additionally, it would avoid the use of preprocessing tools and hence the uncertainty associated with them.

### Limitations of this study

There are multiple limitations in this study. First, only male subjects were used in the study and hence all the findings of this study can be used only in the context of brain abnormalities in male ASD subjects. The findings of this study cannot be generalized to all the ASD population because previous studies have reported that there is a small overlap between the brain abnormalities in ASD males and females. Second, the specific classification models constructed in the AS sub-groups may not have direct practical utility. However, the construction of separate classification models in the sub-groups provide valuable information that the brain features associated with autism are highly dependent upon the autism severity score. This information can be utilized to better understand the brain morphometry in ASD which in turn can aid in clinical diagnosis and treatment.

## Conclusion

This study demonstrated that brain abnormalities in ASD are highly heterogeneous, and the heterogeneity makes the understanding and diagnosis of ASD using brain morphometry, a challenging problem. We showed that the heterogeneity can be mitigated when the demographics and behavioral (DB) measures such as autism severity, VIQ and age are utilized in conjunction with brain morphometric features, hence making the problem of understanding and diagnosis of ASD easier. Utilizing DB measures, the ASD vs. TDC classification success rate was significantly improved. Focusing on relatively homogenous sub-groups of ASD by sub-grouping the subjects according to autism severity, VIQ and age, interesting and valuable relationships between these DB measures and brain morphometry were observed. The heterogeneity in ASD brain morphometric differences demonstrated in this study explains the inconsistent neuroanatomical findings in ASD and the low classification success in large multi-site data. The results of this study suggest that identifying brain biomarkers in relatively homogenous sub-groups by different measures such as age, IQs, severity, sex, handedness etc., compared to identifying markers across the whole ASD population, is easier and also provides better insights on neuroanatomical underpinnings of ASD.

## Supporting Information

S1 Fig Gradient Boosting Machine: Improvement in classification by sub-grouping based on autism severity (AS), age and Verbal IQ (VIQ). The results are similar to that of Random Forest which are presented in Fig 1. A point represents the mean and an error bar represents the one standard deviation of the AUC scores from 10 test folds. **A)** Smaller classes were up-sampled in each training fold to balance the number of ASD & TDC subjects. Sub-grouping improved the classification with the most and least improvements from sub-grouping by AS and age respectively. **B)** Larger classes were down-sampled matching the demographics of the smaller classes. This scheme further improved the classification performance.
(TIFF)

S2 Fig Gradient Boosting Machine (GBM): Important features for classification are variable across sub-groups. Top 10 important features for autism spectrum disorder (ASD) vs. typically developing controls (TDC) classification in each sub-group are presented. Each feature is represented by a colored bar; the length of the bar represents the relative % importance for classification with respect to the top feature. The features have been grouped and color-coded by volume, area, thickness mean, thickness standard deviation, folding index, mean curvature and Gaussian curvature. Before each feature, Cohen’s d and two sample t-test significance (*P*<0.005** and *P*<0.05*) of ASD vs. TDC group difference are presented. Important features for classification were similar to that from random forest presented in Fig 2. The important features highly varied across the sub-groups demonstrating the heterogeneity in ASD brain morphometry.
(TIFF)

S3 Fig Inter and intra sub-groups classification AUC scores. Mean and standard deviations of AUC scores when classification models trained in different sub-groups were tested on each other. The title of each sub-plot represents the sub-group on which the testing was performed. Red data points correspond to when training and testing were performed on the same sub-group (intra-subgroup) and black data points correspond to when training and testing were performed on different sub-groups (inter-subgroup). **Intra-subgroup classification:** A random forest classification model was trained in each sub-group under the 10-fold cross-validation framework. **Inter-subgroup classification:** A random forest classification model trained in each sub-group was tested on 200 bootstrap replications of the test sub-group. Intra sub-groups AUC scores were much larger than inter sub-groups AUC scores in 16 out of 18 comparisons. The AUC scores decreased with the increasing distance between training sub-group and test sub-group.
(PDF)

S4 Fig Random Forest: Important features for classification in sub-groups (10% threshold). Important features for autism spectrum disorder (ASD) vs. typically developing controls (TDC) classification in each sub-group are presented. The features required to have 10% of the total feature importance scores of all the features were considered as important. Each feature is represented by a colored bar; the length of the bar represents the relative % importance for classification with respect to the top feature. The features have been grouped and color-coded by volume, area, thickness mean, thickness standard deviation, folding index, mean curvature and Gaussian curvature. Before each feature, Cohen’s d and two sample t-test significance (*P*<0.005** and *P*<0.05*) of ASD vs. TDC group difference are presented. The important features for classification varied across the sub-groups demonstrating the heterogeneity in ASD brain morphometry. Important features are similar to that from random forest presented in Fig 2 where top 10 features are presented. The important features were dissimilar across the sub-groups.
(PDF)

S5 Fig Random Forest: Important features for classification in sub-groups (25% threshold). Important features for autism spectrum disorder (ASD) vs. typically developing controls (TDC) classification in each sub-group are presented. The features required to have 25% of the total feature importance scores across all the features were considered as important. Each feature is represented by a colored bar; the length of the bar represents the relative % importance for classification with respect to the top feature. The features have been grouped and color-coded by volume, area, thickness mean, thickness standard deviation, folding index, mean curvature and Gaussian curvature. Before each feature, Cohen’s d and two sample t-test significance (*P*<0.005** and *P*<0.05*) of ASD vs. TDC group difference are presented. The important features for classification varied across the sub-groups demonstrating the heterogeneity in ASD brain morphometry. Important features are similar to that from random forest presented in Fig 2 where top 10 features are presented. The important features were dissimilar across the sub-groups.
(PDF)

S6 Fig Classification performance degrades with Autism Severity (AS). Separate classification models were trained for autism spectrum disorder (ASD) subjects with different AS values. A point represents the mean and an error bar represents the one standard deviation of the AUC scores from 10 test folds. AUC scores and number of ASD and TDC subjects are presented below the error bar. Blue line represents the mean AUC vs. mean AS linear model and the shaded region represent the 95% confidence interval of the model. Classification performance decreased with AS according to both random forest and gradient boosting machine classification techniques.
(TIFF)

S1 Table Correlation between the feature importance scores from RF and GBM. (DOCX)
