# Supplementary figures and images for: Divide and Conquer: Sub-Grouping of ASD Improves ASD Detection Based on Brain Morphometry
Source: PLoS One. 2016 Apr 11;11(4):e0153331. doi: 10.1371/journal.pone.0153331 (PMC4827874; doi:10.1371/journal.pone.0153331)

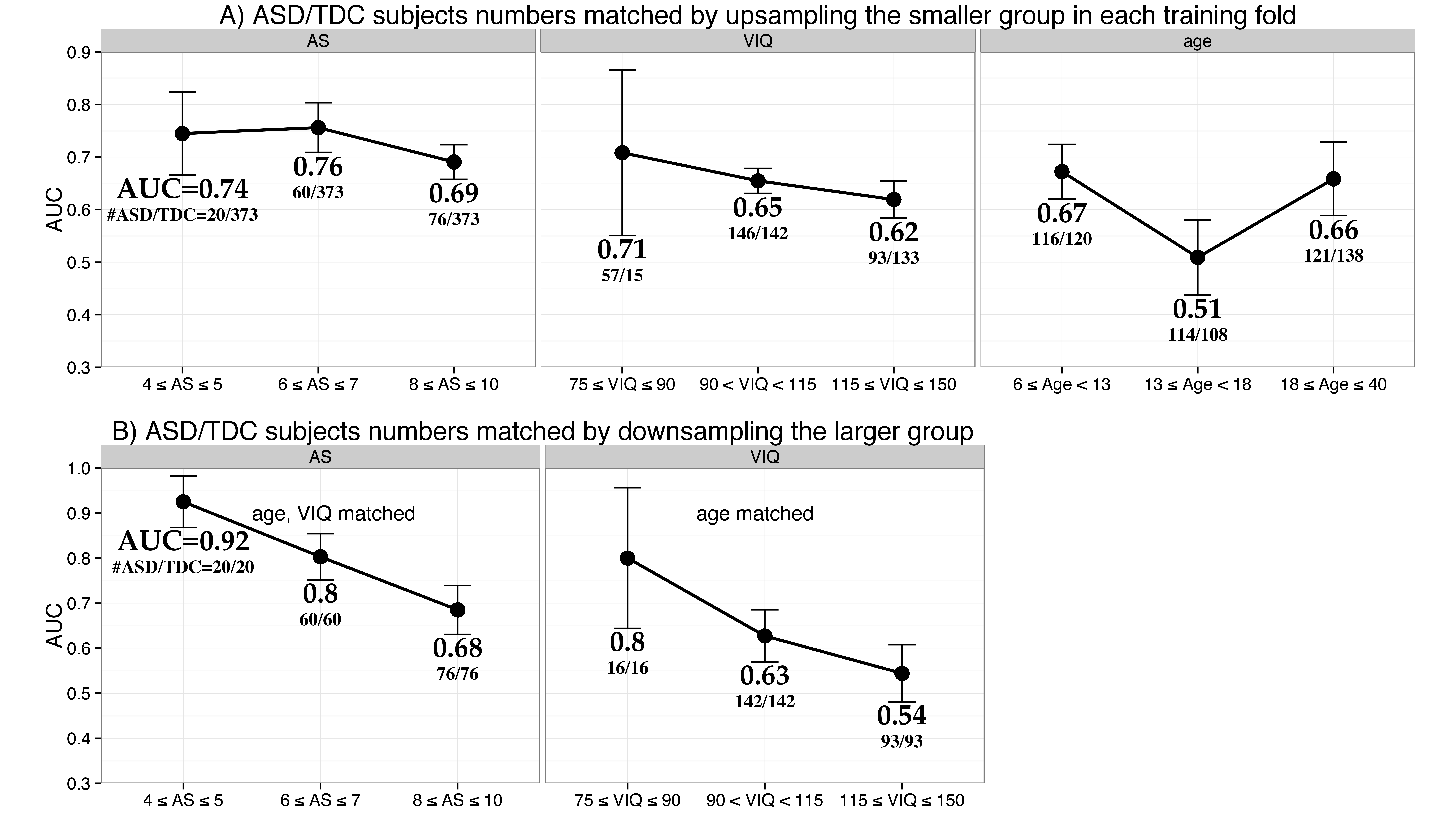

Supplement: S1 Fig — The results are similar to that of Random Forest which are presented in Fig 1. A point represents the mean and an error bar represents the one standard deviation of the AUC scores from 10 test folds. A) Smaller classes were up-sampled in each training fold to balance the number of ASD & TDC subjects. Sub-grouping improved the classification with the most and least improvements from sub-grouping by AS and age respectively. B) Larger classes were down-sampled matching the demographics of the smaller classes. This scheme further improved the classification performance. (TIFF) [file pone.0153331.s001.tiff]

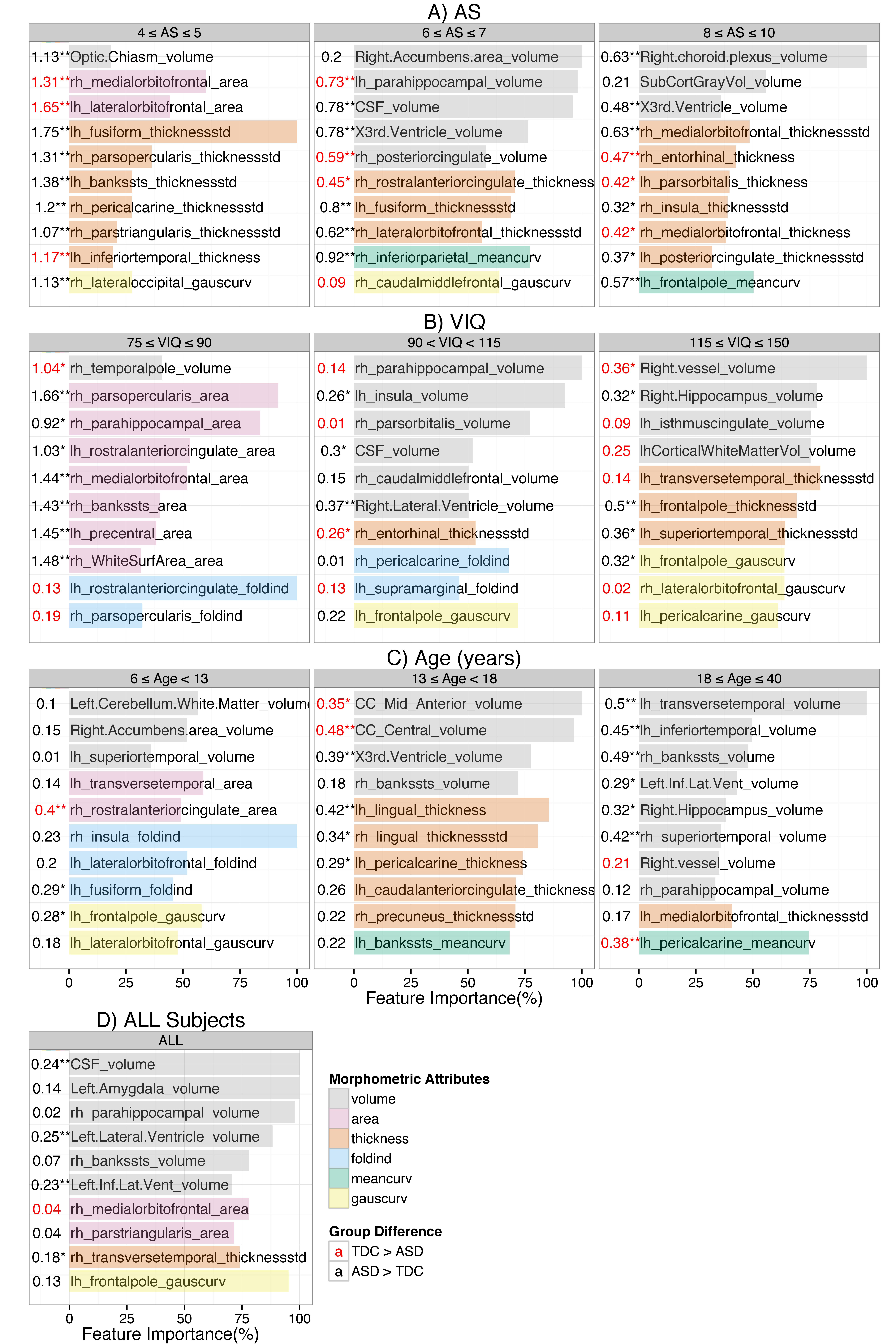

Supplement: S2 Fig — Top 10 important features for autism spectrum disorder (ASD) vs. typically developing controls (TDC) classification in each sub-group are presented. Each feature is represented by a colored bar; the length of the bar represents the relative % importance for classification with respect to the top feature. The features have been grouped and color-coded by volume, area, thickness mean, thickness standard deviation, folding index, mean curvature and Gaussian curvature. Before each feature, Cohen’s d and two sample t-test significance (P<0.005** and P<0.05*) of ASD vs. TDC group difference are presented. Important features for classification were similar to that from random forest presented in Fig 2. The important features highly varied across the sub-groups demonstrating the heterogeneity in ASD brain morphometry. (TIFF) [file pone.0153331.s002.tiff]

A) AS

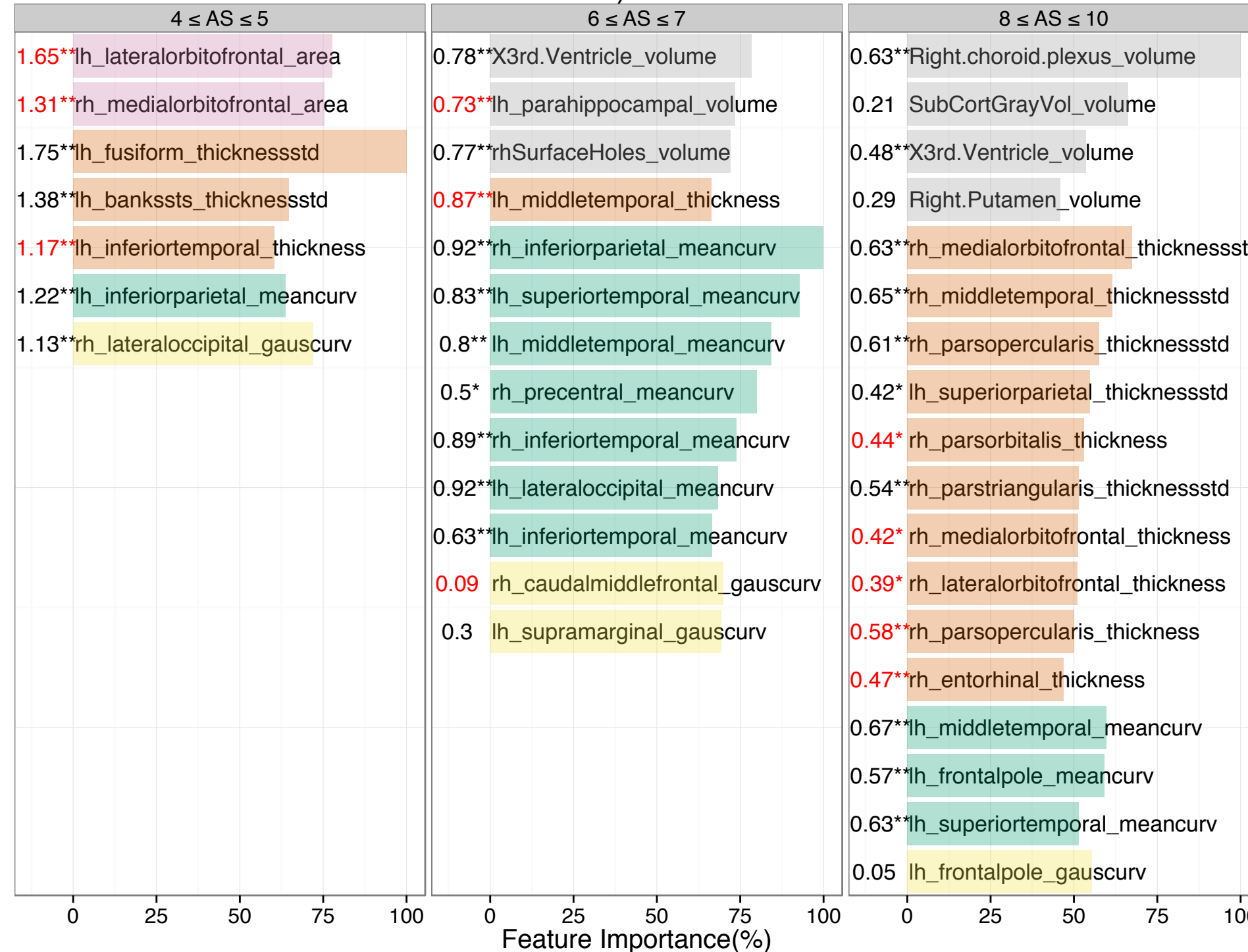

B) VIQ

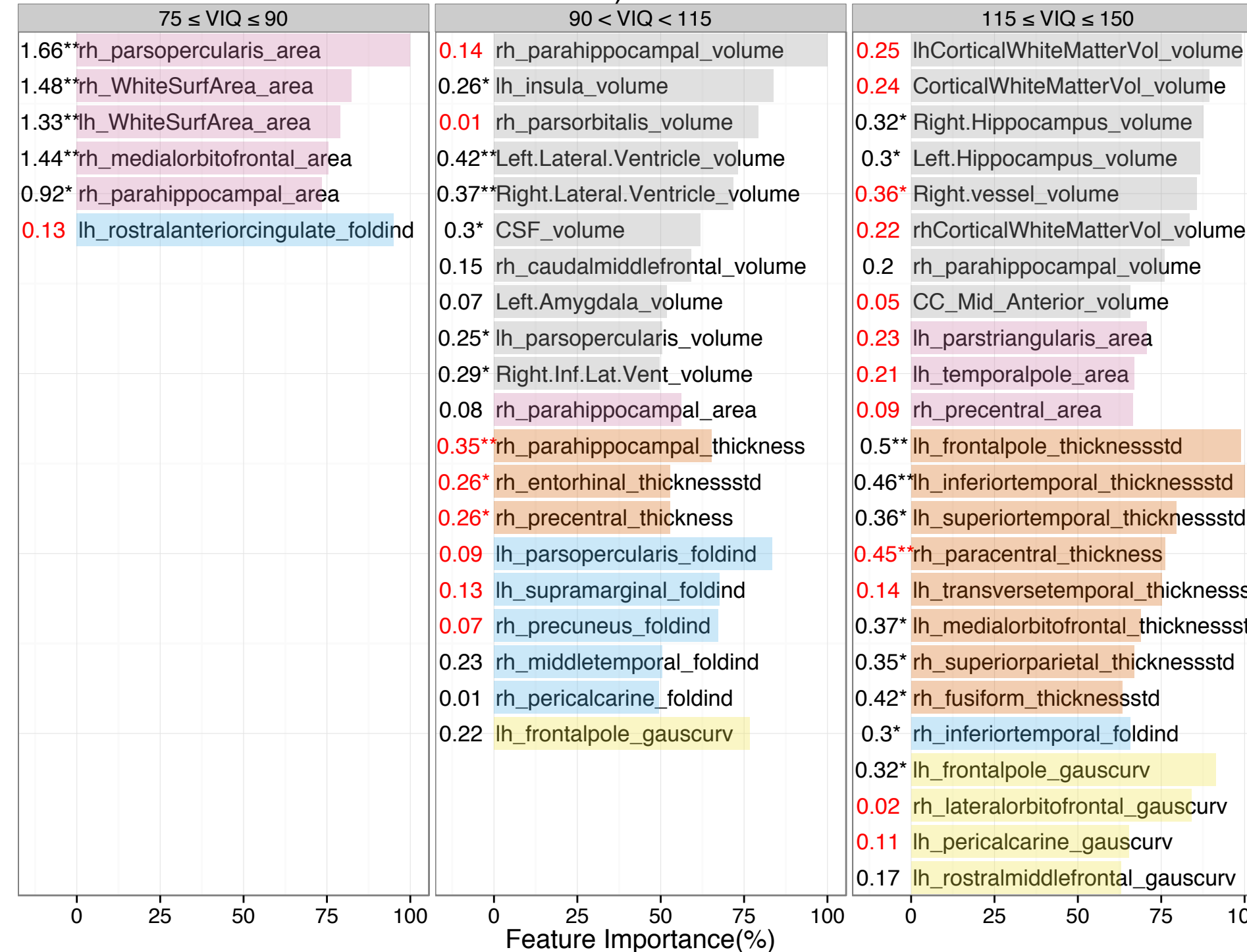

C) Age (years)

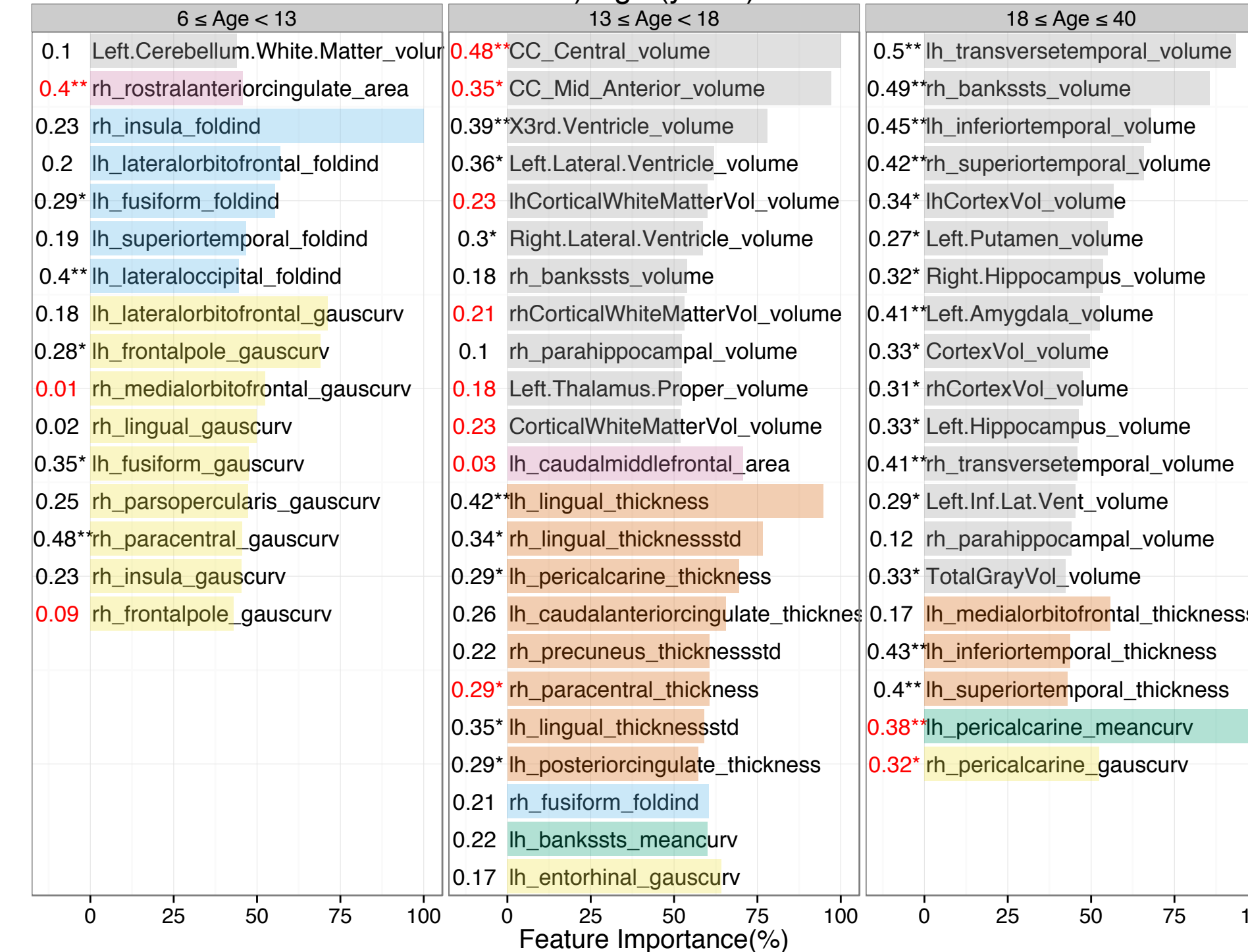

D) ALL Subjects

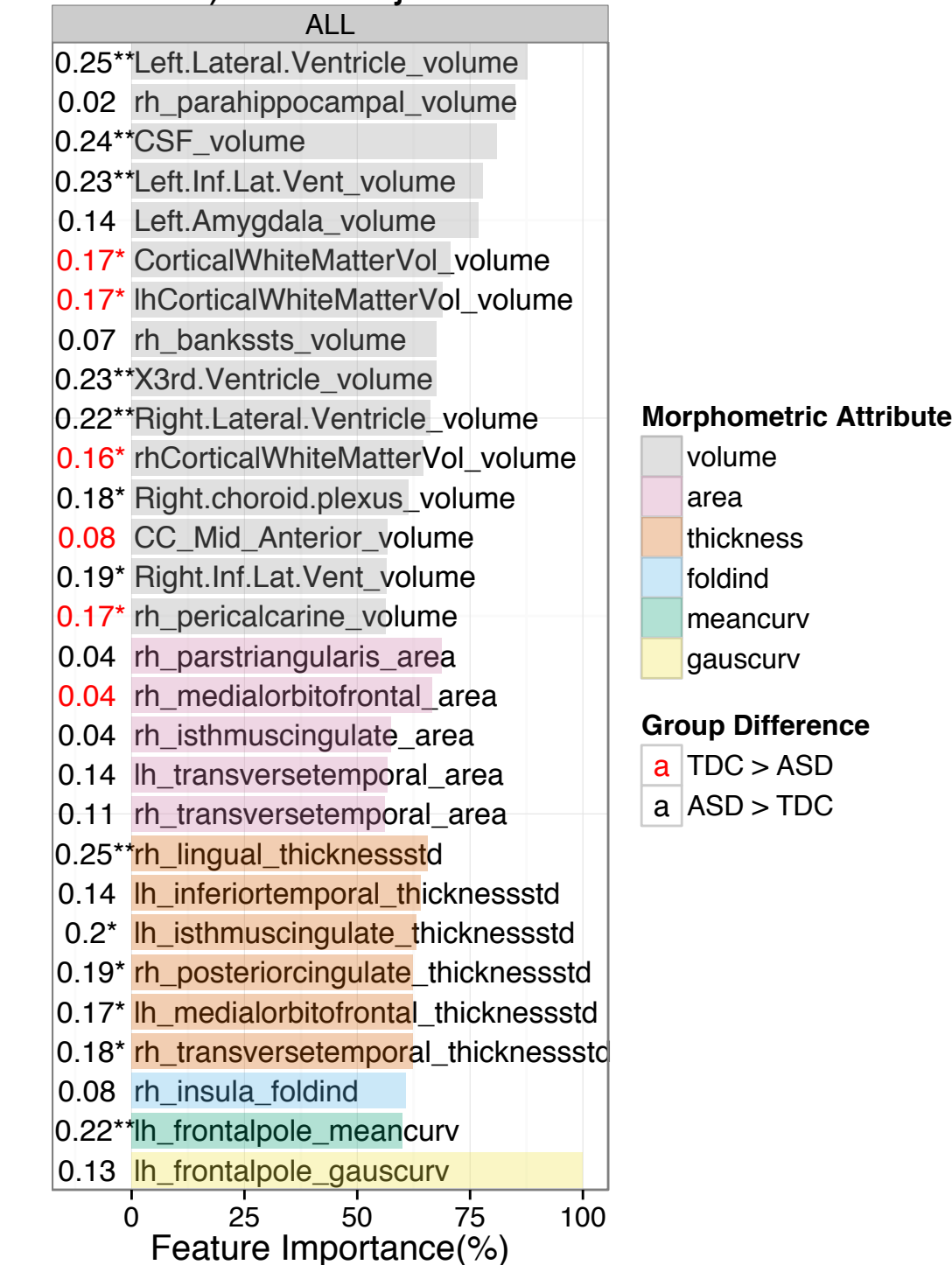

Morphometric Attributes

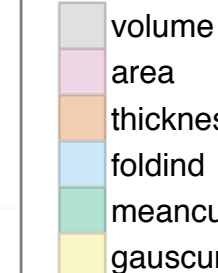

Group Difference

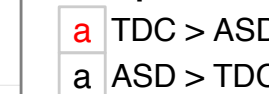

Supplement: S4 Fig — Important features for autism spectrum disorder (ASD) vs. typically developing controls (TDC) classification in each sub-group are presented. The features required to have 10% of the total feature importance scores of all the features were considered as important. Each feature is represented by a colored bar; the length of the bar represents the relative % importance for classification with respect to the top feature. The features have been grouped and color-coded by volume, area, thickness mean, thickness standard deviation, folding index, mean curvature and Gaussian curvature. Before each feature, Cohen’s d and two sample t-test significance (P<0.005** and P<0.05*) of ASD vs. TDC group difference are presented. The important features for classification varied across the sub-groups demonstrating the heterogeneity in ASD brain morphometry. Important features are similar to that from random forest presented in Fig 2 where top 10 features are presented. The important features were dissimilar across the sub-groups. (PDF) [file pone.0153331.s004.pdf]

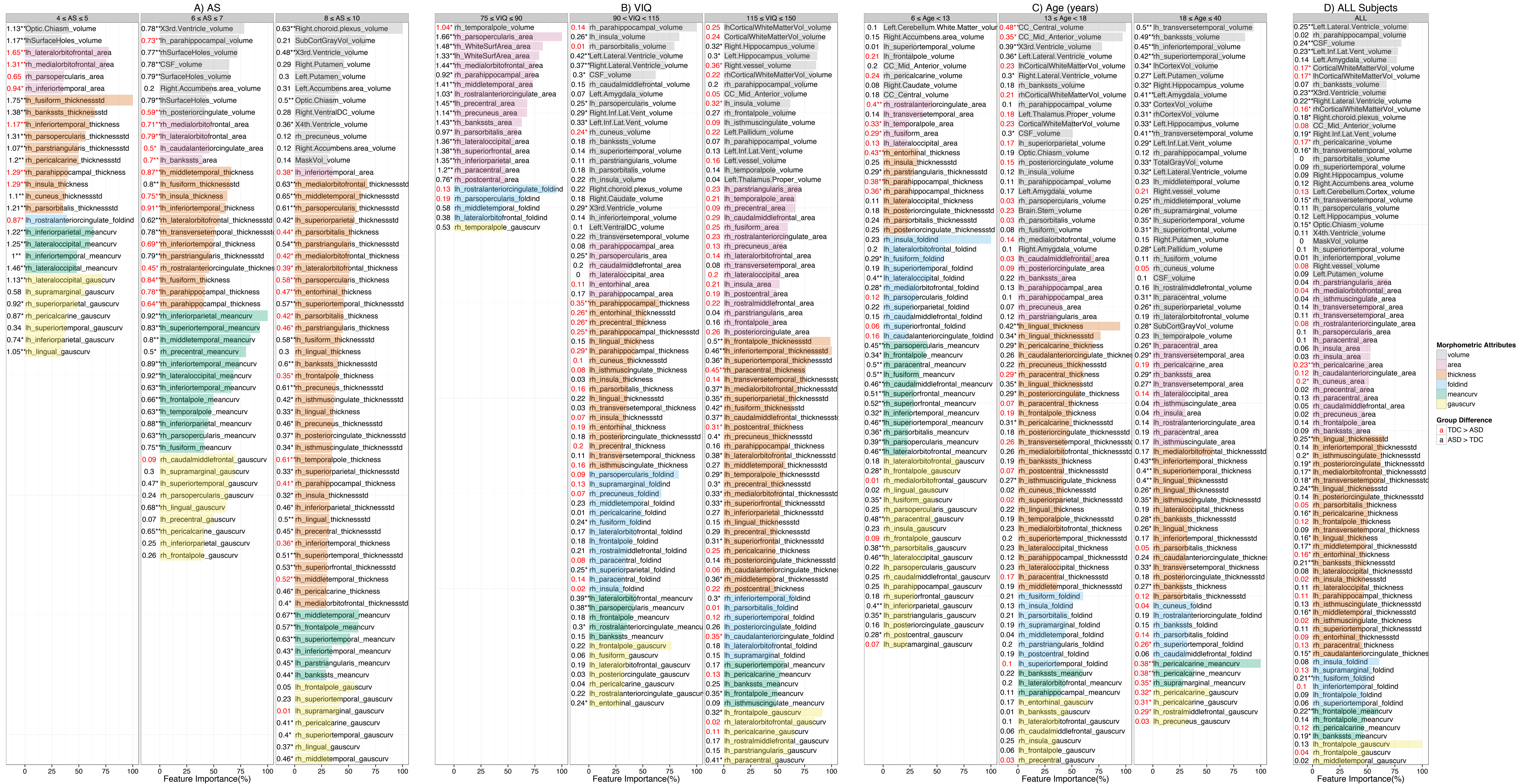

Supplement: S5 Fig — Important features for autism spectrum disorder (ASD) vs. typically developing controls (TDC) classification in each sub-group are presented. The features required to have 25% of the total feature importance scores across all the features were considered as important. Each feature is represented by a colored bar; the length of the bar represents the relative % importance for classification with respect to the top feature. The features have been grouped and color-coded by volume, area, thickness mean, thickness standard deviation, folding index, mean curvature and Gaussian curvature. Before each feature, Cohen’s d and two sample t-test significance (P<0.005** and P<0.05*) of ASD vs. TDC group difference are presented. The important features for classification varied across the sub-groups demonstrating the heterogeneity in ASD brain morphometry. Important features are similar to that from random forest presented in Fig 2 where top 10 features are presented. The important features were dissimilar across the sub-groups. (PDF) [file pone.0153331.s005.pdf]

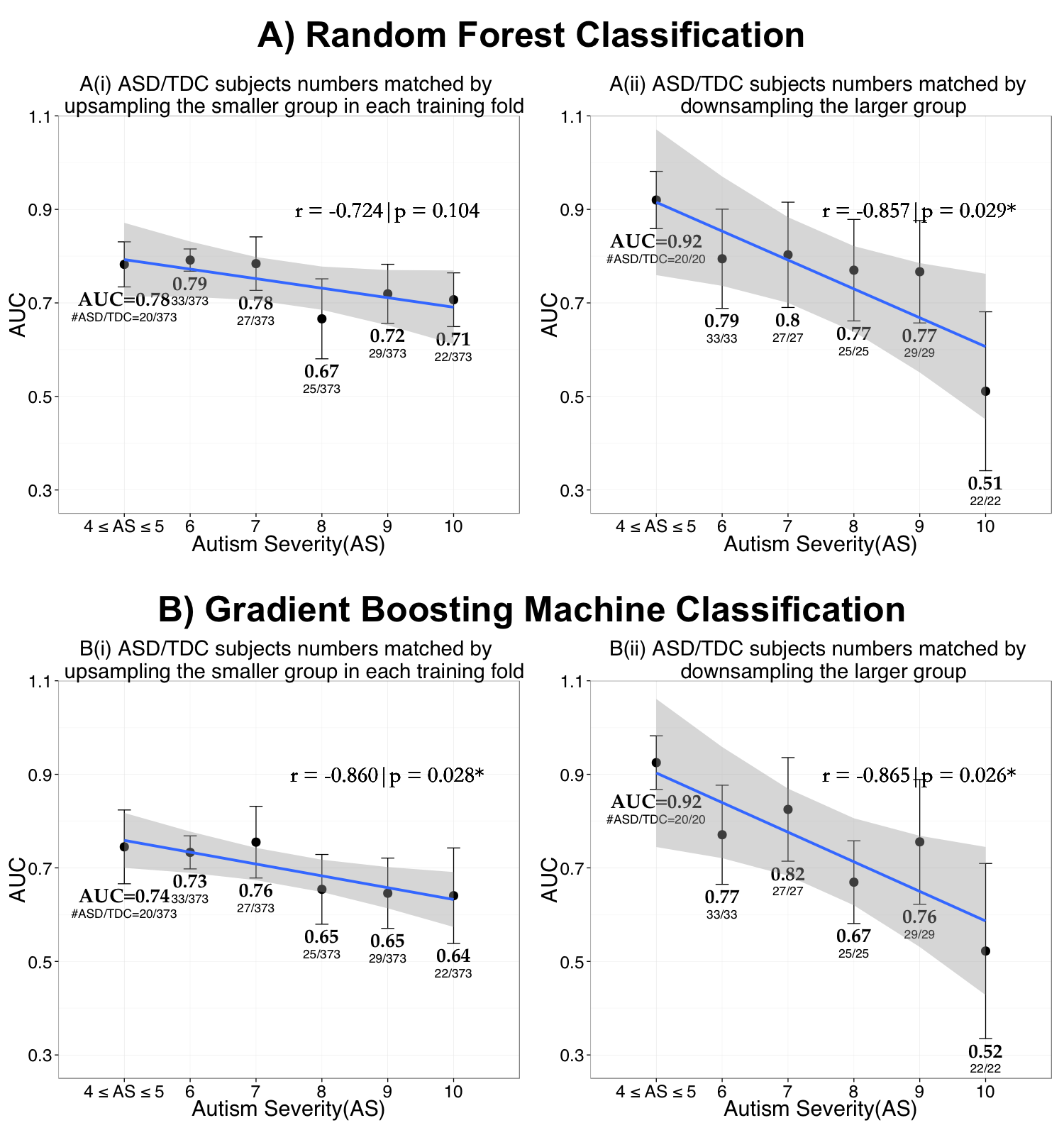

Supplement: S6 Fig — Separate classification models were trained for autism spectrum disorder (ASD) subjects with different AS values. A point represents the mean and an error bar represents the one standard deviation of the AUC scores from 10 test folds. AUC scores and number of ASD and TDC subjects are presented below the error bar. Blue line represents the mean AUC vs. mean AS linear model and the shaded region represent the 95% confidence interval of the model. Classification performance decreased with AS according to both random forest and gradient boosting machine classification techniques. (TIFF) [file pone.0153331.s006.tiff]
