# Supplementary material for: Divide and Conquer: Sub-Grouping of ASD Improves ASD Detection Based on Brain Morphometry
Source: PLoS One. 2016 Apr 11;11(4):e0153331. doi: 10.1371/journal.pone.0153331 (PMC4827874; doi:10.1371/journal.pone.0153331)
Supplement: S3 Fig — Mean and standard deviations of AUC scores when classification models trained in different sub-groups were tested on each other. The title of each sub-plot represents the sub-group on which the testing was performed. Red data points correspond to when training and testing were performed on the same sub-group (intra-subgroup) and black data points correspond to when training and testing were performed on different sub-groups (inter-subgroup). Intra-subgroup classification: A random forest classification model was trained in each sub-group under the 10-fold cross-validation framework. Inter-subgroup classification: A random forest classification model trained in each sub-group was tested on 200 bootstrap replications of the test sub-group. Intra sub-groups AUC scores were much larger than inter sub-groups AUC scores in 16 out of 18 comparisons. The AUC scores decreased with the increasing distance between training sub-group and test sub-group. (PDF) [file pone.0153331.s003.pdf]

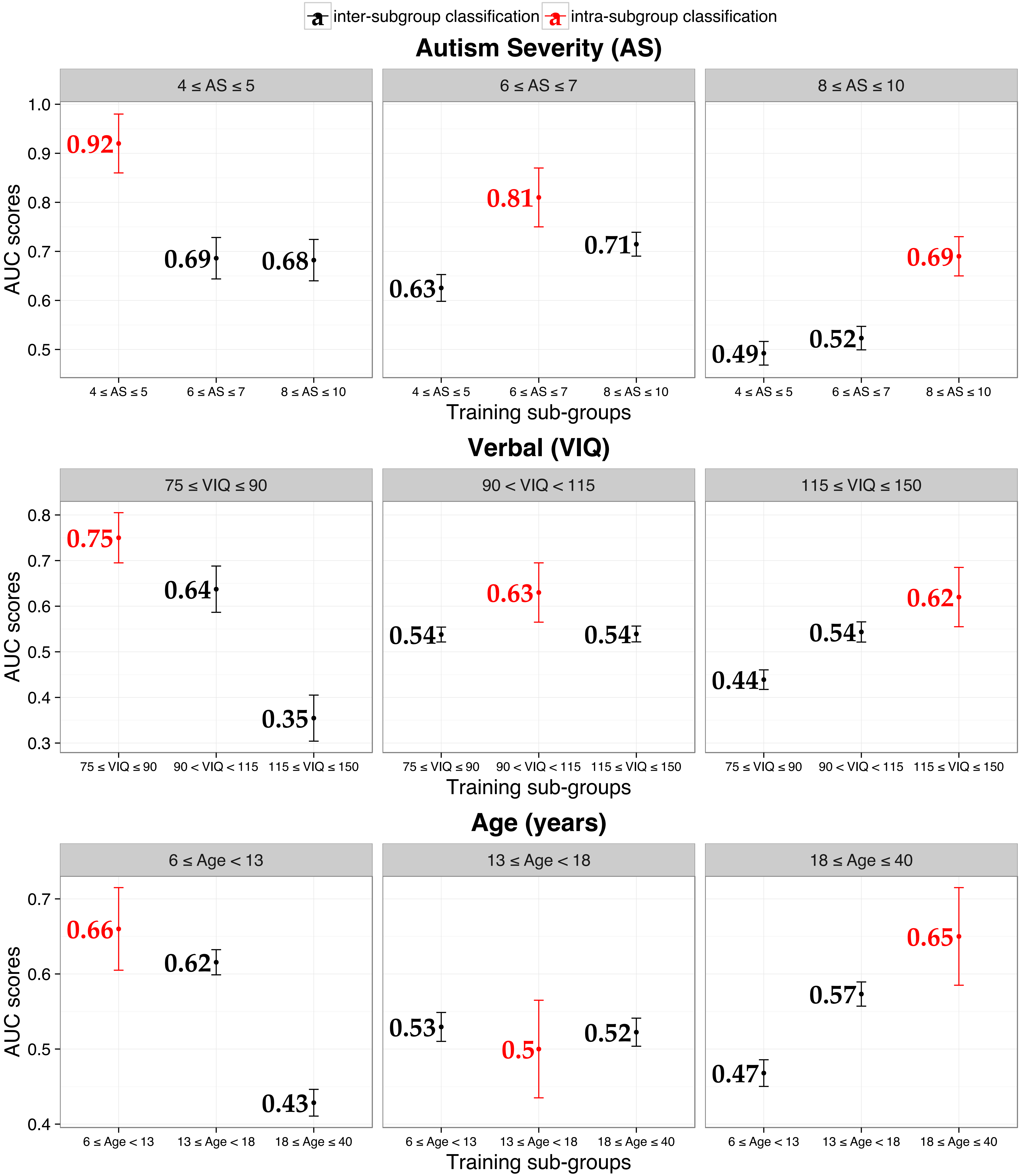

inter-subgroup classification
intra-subgroup classification
Autism Severity (AS)
4 ≤ AS ≤ 5
6 ≤ AS ≤ 7
8 ≤ AS ≤ 10
AUC scores
0.92
0.69
0.68
0.63
0.81
0.71
0.49
0.52
0.69
Training sub-groups
Verbal (VIQ)
75 ≤ VIQ ≤ 90
90 < VIQ < 115
115 ≤ VIQ ≤ 150
0.75
0.64
0.35
0.54
0.63
0.54
0.44
0.54
0.62
Training sub-groups
Age (years)
6 ≤ Age < 13
13 ≤ Age < 18
18 ≤ Age ≤ 40
0.66
0.62
0.43
0.53
0.5
0.52
0.47
0.57
0.65
Training sub-groups
