# Supplementary material for: Divide and Conquer: Sub-Grouping of ASD Improves ASD Detection Based on Brain Morphometry
Source: PLoS One. 2016 Apr 11;11(4):e0153331. doi: 10.1371/journal.pone.0153331 (PMC4827874; doi:10.1371/journal.pone.0153331)
Supplement: S1 Table — (DOCX) [file pone.0153331.s007.docx]

S1 Table. Correlation between the feature importance scores from RF and GBM

|  | **Sub-groups** | **Correlation**  **Coefficient** |
| --- | --- | --- |
| **AS** | *mild* | 0.76 |
|  | *moderate* | 0.77 |
|  | *high* | 086 |
| **VIQ** | *low* | 0.87 |
|  | *normal* | 0.90 |
|  | *high* | 0.88 |
| **Age** | *young* | 0.86 |
|  | *mid* | 0.94 |
|  | *old* | 0.88 |

*All coefficients were statistically significant (p < E-16)*
